# Identifying a distinct fibrosis subset of NAFLD via molecular profiling and the involvement of profibrotic macrophages

**DOI:** 10.1186/s12967-023-04300-6

**Published:** 2023-07-06

**Authors:** Weiwei He, Yinxiang Huang, Xiulin Shi, Qingxuan Wang, Menghua Wu, Han Li, Qiuhong Liu, Xiaofang Zhang, Caoxin Huang, Xuejun Li

**Affiliations:** 1 Department of Endocrinology and Diabetes, The First Affiliated Hospital of Xiamen University, School of Medicine, Xiamen University, Xaimen, China; 2 Xiamen Diabetes Institute, The First Affiliated Hospital of Xiamen University, Xiamen, China; 3 Fujian Provincial Key Laboratory of Translational Medicine for Diabetes, Xiamen, China

**Keywords:** Non-alcoholic fatty liver disease, Fibrosis subtype, Transcriptomic signatures, Non-negative matrix factorization, Profibrotic macrophages

## Abstract

**Background:**

There are emerging studies suggesting that non-alcoholic fatty liver disease (NAFLD) is a heterogeneous disease with multiple etiologies and molecular phenotypes. Fibrosis is the key process in NAFLD progression. In this study, we aimed to explore molecular phenotypes of NAFLD with a particular focus on the fibrosis phenotype and also aimed to explore the changes of macrophage subsets in the fibrosis subset of NAFLD.

**Methods:**

To assess the transcriptomic alterations of key factors in NAFLD and fibrosis progression, we included 14 different transcriptomic datasets of liver tissues. In addition, two single-cell RNA sequencing (scRNA-seq) datasets were included to construct transcriptomic signatures that could represent specific cells. To explore the molecular subsets of fibrosis in NAFLD based on the transcriptomic features, we used a high-quality RNA-sequencing (RNA-seq) dataset of liver tissues from patients with NAFLD. Non-negative matrix factorization (NMF) was used to analyze the molecular subsets of NAFLD based on the gene set variation analysis (GSVA) enrichment scores of key molecule features in liver tissues.

**Results:**

The key transcriptomic signatures on NAFLD including non-alcoholic steatohepatitis (NASH) signature, fibrosis signature, non-alcoholic fatty liver (NAFL) signature, liver aging signature and TGF-β signature were constructed by liver transcriptome datasets. We analyzed two liver scRNA-seq datasets and constructed cell type-specific transcriptomic signatures based on the genes that were highly expressed in each cell subset. We analyzed the molecular subsets of NAFLD by NMF and categorized four main subsets of NAFLD. Cluster 4 subset is mainly characterized by liver fibrosis. Patients with Cluster 4 subset have more advanced liver fibrosis than patients with other subsets, or may have a high risk of liver fibrosis progression. Furthermore, we identified two key monocyte-macrophage subsets which were both significantly correlated with the progression of liver fibrosis in NAFLD patients.

**Conclusion:**

Our study revealed the molecular subtypes of NAFLD by integrating key information from transcriptomic expression profiling and liver microenvironment, and identified a novel and distinct fibrosis subset of NAFLD. The fibrosis subset is significantly correlated with the profibrotic macrophages and M2 macrophage subset. These two liver macrophage subsets may be important players in the progression of liver fibrosis of NAFLD patients.

**Supplementary Information:**

The online version contains supplementary material available at 10.1186/s12967-023-04300-6.

## Introduction

Non-alcoholic fatty liver disease (NAFLD) is a common liver disease characterized by abnormal lipid deposition in the liver [1, 2]. The prevalence of NAFLD in general population is about 20–30%, and is approximately 80% in patients with type 2 diabetes mellitus (T2DM) [3, 4]. It can significantly increase the risk of diabetic complications and cardiovascular disease, and has now become the leading cause of liver disease worldwide [3, 4]. In China, NAFLD is growing rapidly, and has become the commonest liver disease [5]. NAFLD mainly includes non-alcoholic fatty liver (NAFL) and non-alcoholic steatohepatitis (NASH) [6, 7], and about 20–25% of NASH patients may develop cirrhosis or even hepatocellular carcinoma [6]. NASH is a critical turning point in the progression of NAFLD, and in addition to abnormal lipid deposition in the liver, NASH is associated with pathological changes such as ballooning of hepatocytes and inflammation of liver [7]. The treatment of NASH still mainly relies on lifestyle intervention, and liver transplantation is the only effective treatment for patients with severe fibrosis and liver failure [8]. Therefore, there is an urgent need to further explore the pathogenesis of NAFLD and to innovative treatment options.

Recent studies have shown that fibrosis is the key process in NAFLD progression [9]. In NAFLD patients, fibrosis stage is the most significant predictor of cirrhosis, liver transplantation, and liver-related death, and the risk of liver-related death rises exponentially with stage [10]. Our previous study of integrated transcriptomic analyses identified fibrosis-related pathways as the most crucial role in the progression of NASH [11]. Some studies have suggested that fibrosis is a pathological process which can be independent of the pathological features of NAFLD such as energy disturbance and lipotoxic liver injury [12–15]. Moreover, there are emerging studies suggesting that NAFLD is a heterogeneous disease with multiple etiologies and multiple molecular phenotypes exist in NAFLD [16, 17]. Therefore, a better understanding of the molecular phenotypes of NAFLD especially the fibrosis progression is critical and may be helpful to the development of phenotype-based personalized therapy in the future. The main pathological changes and core pathogenic mechanisms involved in different NAFLD patients may also be different, so the accurate therapeutic targets and methods may also be different. Since different subtypes of patients may have different therapeutic approaches and clinical outcomes, accurately defining the molecular subtypes of NAFLD is a prerequisite for achieving precise treatment of NAFLD. The molecular subtypes of NAFLD are still unclear and the roles of macrophage subsets in the subtypes of NAFLD have also not been defined. In this study, we aimed to explore molecular phenotypes of NAFLD with a particular focus on the fibrosis phenotype and also aimed to explore the changes of macrophage subsets in the fibrosis subset of NAFLD. We analyzed the altered key transcriptomic features related to liver immune microenvironment and fibrosis progression, and then used non-negative matrix factorization (NMF) method to analyze the heterogeneity and molecular phenotypes of NAFLD. The transcriptomic and immune features of the fibrosis phenotype in NAFLD were also explored.

## Methods

### Liver transcriptome datasets of NAFLD patients

To assess the transcriptomic alterations of key factors in NAFLD and fibrosis progression, we included different transcriptomic datasets of liver tissues through the Gene Expression Omnibus (GEO) database. To assess transcriptomic alterations in the liver of NAFL patients with predominantly steatosis, we used those human liver transcriptomic datasets containing NAFL patients and healthy controls to establish a transcriptomic signature that can assess the degree of steatosis in the liver of NALFD patients. For datasets of NAFL liver tissues, those meeting the following inclusion criteria were used: (1) Transcriptome data of liver tissues; (2) more than 30 differentially expressed genes (DEGs) were found to be differently expressed between the two groups; (3) had genome expression profiles; (4) samples had to include both normal liver tissues and NAFL liver tissues. To assess liver aging via transcriptomic profile, we used four human liver transcriptomic datasets containing older and younger controls to establish a transcriptomic signature that can assess the degree of liver aging. In our study, we used the raw transcriptomic data and analyses methods used in our study were different from that in the original studies. The information of relevant published studies had been added in the Additional file 2: Table S2. In addition, to assess the impact of liver immune microenvironment on NAFLD and fibrosis, two single-cell RNA sequencing (scRNA-seq) datasets were included to construct transcriptomic signatures that could represent HSCs, hepatocytes, endothelial cells, cholangiocytes, Kupffer cells, macrophages, B cells, T cells and NK cells. To explore the molecular subtypes of fibrosis in NAFLD based on the transcriptomic features, we used a high-quality RNA-sequencing (RNA-seq) dataset of liver tissues from NAFLD patients.

### Differential expression analyses and robust rank aggregation (RRA) analysis

For the integration analysis, we first need to analyze the differential expression of genes in each dataset. In the analysis of the microarray transcriptome dataset, “limma” package of R were used to analyze the DEGs of the microarray dataset [18]. For the analysis of RNA-seq transcriptome dataset, we used “DESeq2” for differential expression analysis [19]. RRA was used to integrate the results of differential analysis of multiple datasets, and was performed using the R package of “RobustRankAggreg” [20]. Absolute values of log2 fold change (log2FC) larger than 0.30 for genes with adjusted P values less than 0.05 in the RRA analysis were considered statistically significant.

### scRNA-seq analysis

Human liver tissues were analyzed using scRNA-seq to discover the hallmark genes for each cell type in the liver. We used the “Seurat” package for the analysis of scRNA-seq data [21]. We identified the types of cell subsets with both SingleR and the expression patterns of some widely used marker genes for specific cells [22]. Those genes highly expressed in one specific cell subset but not in others were selected as potential transcriptomic signatures for this subset.

### Gene set variation analysis (GSVA)

To evaluate the changes of molecular features in each sample, GSVA analysis was performed with the gene sets of those features such as fibrosis and liver microenvironment cells [23]. The GSVA enrichment score could reflect the degree in the increment or reduction of those molecular features in the liver tissues. This part of the study was analyzed using the “GSVA” of the R package.

### Gene set enrichment analysis (GSEA)

GSEA was performed to assess the difference of molecular features between cases and controls based on the transcriptomic data [24]. Normalized enrichment score (NES) absolute value greater than 1 and false discovery rate (FDR) q-value less than 0.25 were used as the criteria in defining a significant difference. GSEA software (version 3.0, The GSEA/MSigDB Team, Broad Institute, USA) was used in our study.

### Identification of the molecular subsets of NAFLD with NMF method

The NMF method is a widely used methods for defining molecular subsets and is effective in revealing the heterogeneity of complex diseases [25–27]. In this study, NMF was used to analyze the molecular subsets of NAFLD based on the GSVA enrichment scores of key molecule features in liver tissues. First, we calculated the GSVA enrichment scores of the key transcriptomic signatures in each sample, and GSVA enrichment scores were used as the feature data for NMF clustering analysis. The key transcriptomic signatures used mainly included fibrosis-related transcriptomic signatures, NAFL transcriptomic signature, liver immune microenvironment-related transcriptomic signatures, and aging-related transcriptomic signature. This part of the study was conducted by “NMF” of the R package.

### Experimental animals and histological examination

Male DBA/2J mice at 8-weeks were purchased from Beijing Vital River Laboratory Animal Technology Co., Ltd. Mice were housed at appropriate temperature and humidity and provided with adequate water and food. Experiments were started after 2 weeks of acclimatization. Animal experiments in our study were carried out in accordance with animal ethical guidelines. This study was authorized by the Ethics Committee of Xiamen University’s First Affiliated Hospital (Ethics Number: 2021J011344). DBA/2J mice were randomly divided into CCl4-induced liver fibrosis group and control group, with 6 mice in each group. Mice from the CCl4-induced liver fibrosis group received intraperitoneal injections of 10% CCl4 olive oil solution twice per week for a total of 6 weeks. To identify the morphological alterations, the paraffin-embedded mouse liver tissues were cut into 5 μm thick slices and stained with hematoxylin and eosin (H&E). Masson staining was used to determine the degree of tissue fibrosis.

### Immunohistochemistry

Immunohistochemical staining was used to detect the expression levels of Collagen 1, α-Smooth muscle actin (α-SMA) and Triggering receptor expressed on myeloid cells 2 (Trem2) in the liver tissues of mice. Anti-Collagen 1 antibody (1:200, 72026T, CST), anti-α-SMA antibody (1:200, ab5694, Abcam) and anti-Trem2 antibody (1:200, M079792S, Abmart) were used for immunohistochemical staining. Paraffin sections were deparaffinized in xylene and soaked in anhydrous alcohol, 95% alcohol, and 70% alcohol, incubated with hydrogen peroxide blocking solution at room temperature for thermal antigen repair, followed by incubation with 5% bovine serum albumin (BSA) blocking solution at room temperature. After an overnight incubation with the primary antibody at 4 °C, the sections were then treated with the secondary antibody, and finally the sections were counterstained with hematoxylin. Finally, Image J software (National Institutes of Health, Bethesda, MD, USA) was used to measure the intensity of staining.

### Statistical analysis

The Mann–Whitney U test was used to examine the differences in gene expression levels between groups. The unpaired *t*-test was used to compare the enrichment scores of GSVA between groups. We also used Receiver Operating Characteristic (ROC) analysis to further validate the diagnostic efficacy of transcriptomic signatures in identifying the fibrosis subset of NAFLD. P < 0.05 was considered statistically significant. R software (Version 3.6.1, The R Foundation) was used in data analyses.

## Results

### Establishment and validation of key transcriptomic signatures

In order to explore the molecular subtypes of NAFLD by the molecular expression profile of the liver samples, we needed to establish the key transcriptomic signatures of NAFLD. We mainly focused on the molecular subtypes of NAFLD associated with fibrosis, so the included transcriptomic signatures were mainly those closely related to fibrosis or NASH progression. Fibrosis-related transcriptomic signatures were those genes associated with fibrosis progression and NASH progression which have been identified in our previously published study [11], and the transcriptomic signature of transforming growth factor beta (TGF-β) activated HSCs. In addition to the fibrosis-related transcriptomic signatures, we also used NAFL, liver immune microenvironment transcriptomic signatures, and aging-related transcriptomic signature, all of which have important roles in the progression of NAFLD or fibrosis.

In our previously published study, by RRA analysis of 12 transcriptome datasets comparing NASH and NAFL patients, we found 116 genes significantly up-regulated in NASH patients and constructed a NASH progression-associated transcriptomic signature (NASH signature). We also identified 78 genes significantly up-regulated in patients of NAFLD with advanced fibrosis by RRA analysis of 5 liver transcriptome datasets and constructed the NAFLD fibrosis transcriptomic signature (Fibrosis signature) (Additional file 2: Table S1). Therefore, we successfully established transcriptomic signatures that can assess the severity of NASH and liver fibrosis.

To construct the NAFL transcriptomic signature (NAFL signature), we searched the GEO database and included a total of 6 transcriptome datasets comparing liver tissues of NAFL patients and healthy controls, including GSE48452, GSE66676, GSE89632, GSE126848, GSE130970 and GSE135251. We first analyzed the DEGs between groups for each dataset, and then integrated the results of each dataset by RRA analysis. The top 30 genes that were highly expressed in NAFL patients were selected to construct the NAFL signature (Additional file 1: Fig. S1A, Additional file 2: Table S1 and S2). In the validation study, NAFL patients had significantly higher GSVA enrichment score of NAFL signature than healthy controls (P < 0.05; Additional file 1: Fig. S1B), and ROC analysis showed that NAFL signature could effectively identify NAFL patients, indicating that NAFL signature had an accurate diagnostic efficacy in identifying NAFL (Additional file 1: Fig. S1C). Therefore, we successfully established a transcriptomic signature that can effectively identify patients with NAFL.

Accumulating studies have confirmed that one of the key risk factors for the development of liver fibrosis is liver aging, so we also established a transcriptomic signature that could be used to assess the degree of liver aging [28–30]. To construct a liver aging-related transcriptomic signature (Liver aging signature), we included 4 transcriptomic datasets comparing the livers of older individuals with younger individuals by searching the GEO database, including GSE61260, GSE107037, GSE133815 and GSE183915. First, we analyzed each dataset for DEGs between the two groups, and then integrated the results of each dataset by RRA analysis. The top 30 genes that were highly expressed in liver tissues of aged individuals were used to constitute the Liver aging signature (Additional file 1: Fig. S2A, Additional file 2: Table S1 and S2). The GSVA enrichment score for the Liver aging signature was higher in the livers of older individuals than in younger individuals (P = 0.06; Additional file 1: Fig. S2B). Linear correlation analysis revealed a significant correlation between the GSVA enrichment score of Liver aging signature in liver tissue and the real age of the included individuals (r = 0.39, P < 0.0001; Additional file 1: Fig. S2C), suggesting that Liver aging signature could be used to assess the degree of aging in the liver. Therefore, we successfully established a transcriptomic signature that can assess the degree of liver aging.

To construct a transcriptomic signature of hepatic stellate cells (HSCs) activation, we analyzed the RNA-seq data of 6 TGF-β-stimulated HSCs and 6 control HSCs in the GSE148849 dataset, and selected the top 30 genes that were significantly highly expressed in TGF-β-stimulated HSCs to constitute the transcriptomic signature of HSCs activation (TGF-β signature) (Additional file 1: Fig. S3A and B, Additional file 2: Table S1 and S2). In TGF-β stimulated HSCs group, the GSVA enrichment score of the TGF-β signature was considerably higher than control group (P < 0.0001; Additional file 1: Fig. S3C), indicating that TGF-β signature could accurately assess the activation of HSCs. Therefore, we successfully established a transcriptomic signature that can be used to assess the degree of HSCs activation.

**Fig. 1:**
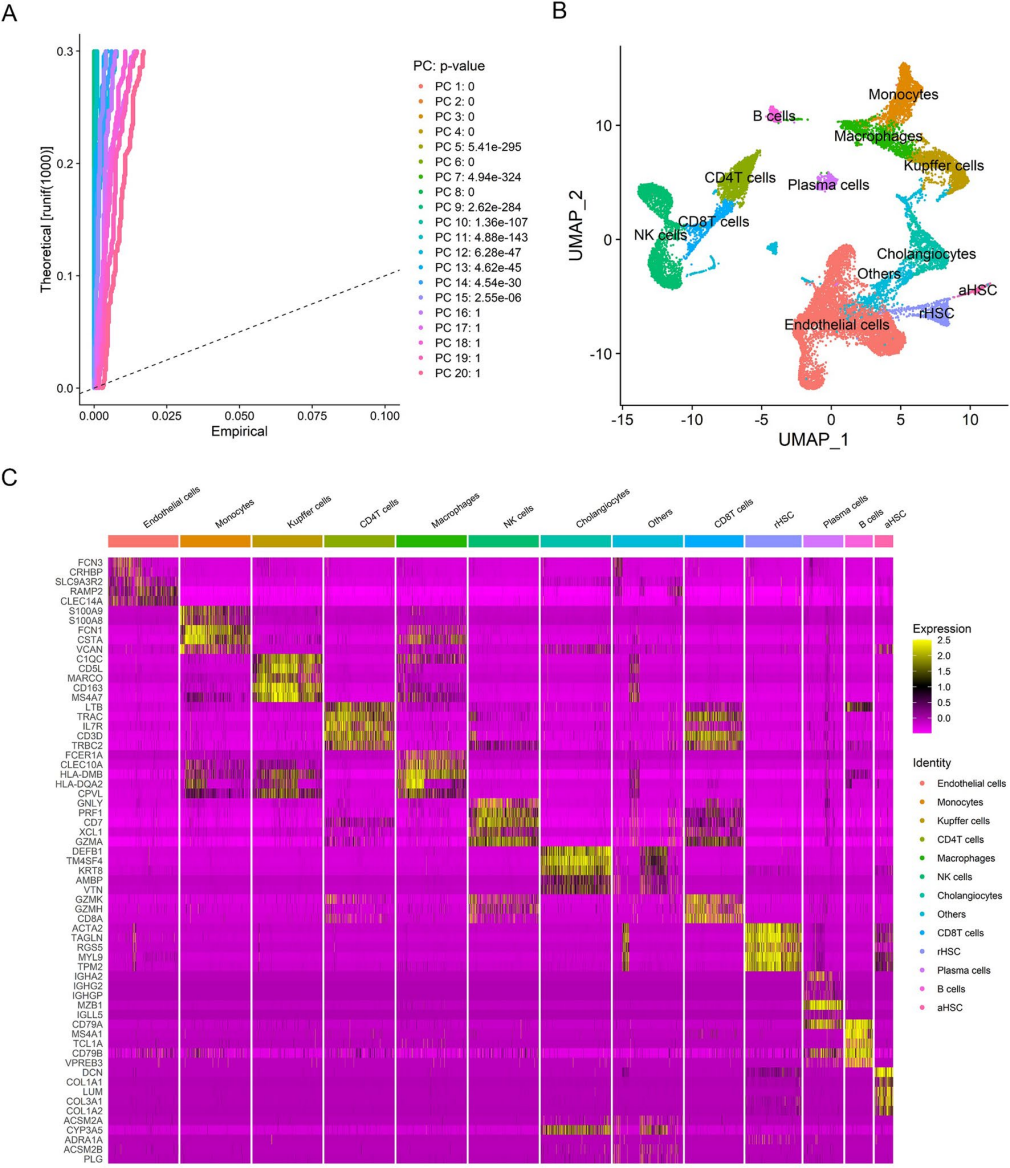
Analyses of liver single cell transcriptomic data in GSE136103. **A** Determination of the statistical significance of PCA scores with JackStraw method. **B** UMAP plot of cell clusters in the liver tissues. **C** Heatmap showing the expression of key genes in different cell clusters of liver tissues

**Fig. 2:**
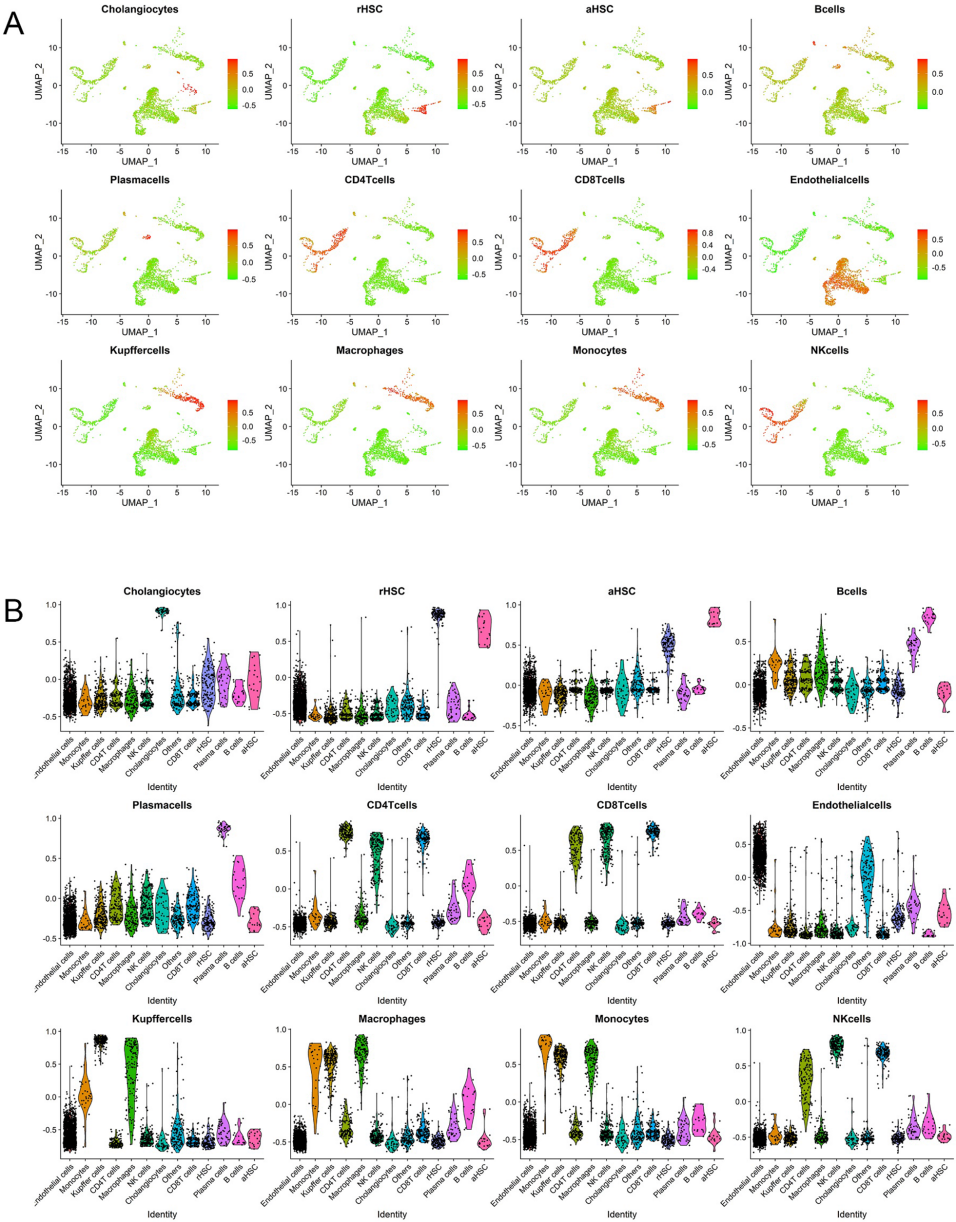
Establishment and validation of liver microenvironment transcriptomic signatures by single cell RNA-sequencing analysis. **A** GSVA revealed that cell type-specific signatures were obviously enriched in each cluster. **B** Comparison of the difference in the enrichment scores of cell signatures in each cluster by violin plots

Since distinct cells in the liver such as hepatocytes, endothelial cells and immune cells can play roles in the progression of NAFLD and fibrosis, we also need to explore the subtype of NAFLD associated fibrosis based on those liver microenvironment-related transcriptomic signatures [31–38]. We analyzed two liver scRNA-seq datasets (GSE136103 and GSE174748) and constructed cell type-specific transcriptomic signatures based on the genes that each cell subset significantly expressed. The cell types in liver tissue mainly included resting HSCs (rHSCs), activated HSCs (aHSCs), hepatocytes, endothelial cells, cholangiocytes, Kupffer cells, macrophages, monocytes, B cells, CD4^+^ T cells, CD8^+^ T cells and NK cells (Figs. 1 and 2, Additional file 2: Table S1 and S2). In the analysis of the GSE136103 dataset, we first used CD45^−^ cells from this dataset, and a total of 8 liver samples with high-quality single-cell transcriptomes were included, namely GSM4041157, GSM4041162, GSM4041167, GSM4041156, GSM4041165, GSM4041159, GSM4041163, and GSM4041151. scRNA-seq analysis was subsequently performed after integration by the SCTtransform method in R software, and by using the UMAP algorithm, these cells were divided into 20 clusters (Fig. 1A and B). We used scRNA-seq analysis to identify the top 30 genes that are highly expressed in particular cells and to establish the transcriptome signatures that could be used to identify the corresponding cell subsets (Fig. 1C). We assessed the enrichment of these transcriptomic signatures in GSM4041156 liver samples by GSVA in order to confirm if those transcriptomic signatures could be utilized to characterize the respective cell subpopulations. The results showed that each transcriptomic signature was significantly enriched in the respective cellular subpopulations, suggesting that each transcriptomic signature could represent the corresponding cell subsets well (Fig. 2A and B). Since the number of hepatocytes in the GSE136103 scRNA-seq dataset was extremely small, the genes highly expressed in hepatocytes could not be accurately analyzed. To better construct a hepatocyte-related transcriptomic signature, a signal-nuclear RNA sequencing (snRNA-seq) dataset (GSE174748) were used (Additional file 1: Fig. S4A and SB). By snRNA-seq analysis, we identified the signature genes expressed by hepatocytes and constructed the hepatocyte transcriptomic signature (Hepatocyte signature) (Additional file 1: Fig. S4C, Additional file 2: Table S1 and S2). Therefore, we successfully established liver microenvironment-related transcriptomic signatures, including rHSCs, aHSCs, hepatocytes, endothelial cells, cholangiocytes, Kupffer cells, macrophages, monocytes, B cells, CD4^+^ T cells, CD8^+^ T cells, and NK cells.

To sum up, we established characteristic transcriptomic signatures including liver fibrosis, NAFL signature, liver immune microenvironment and liver aging signature by integrating human liver tissue transcriptome datasets and liver scRNA-seq datasets.

### Identifying a distinct fibrosis subset of NAFLD via molecular profiling

**Fig. 3:**
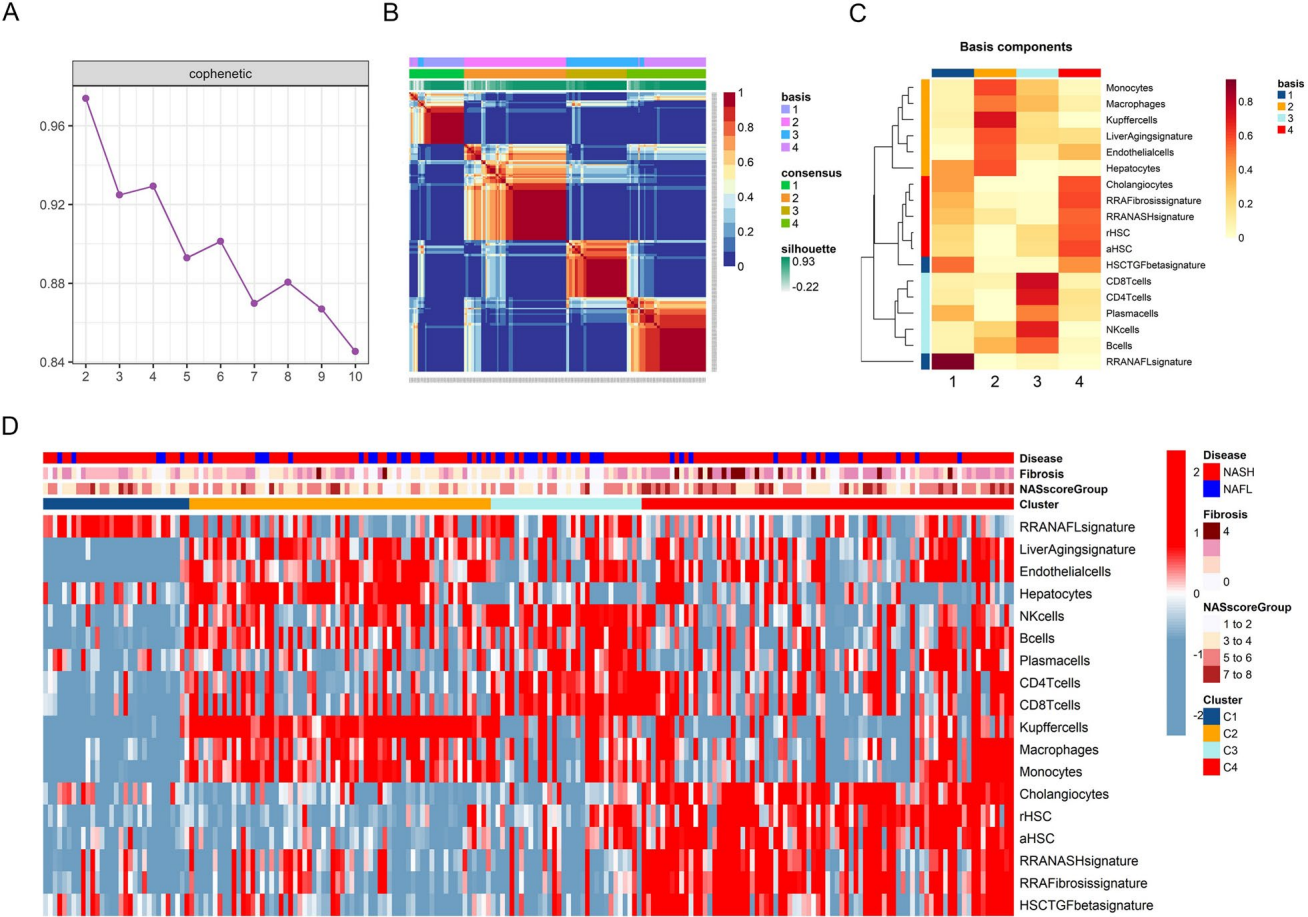
NMF clustering of 206 NAFLD liver samples from GSE135251. **A** Plot of the cophenetic correlation coefficients associated with different numbers of clusters. **B** Consensus map of NMF clustering. **C** Heatmap of mean levels of molecular features in different molecular subsets. **D** Heatmap of molecular features in different molecular subsets in each sample

In order to accurately explore the molecular subsets of NAFLD based on the molecular profiling of the liver samples, we used a high-quality NAFLD liver RNA-seq dataset (GSE135251), which included liver transcriptome data from a total of 206 NAFLD patients and 10 healthy individuals. We used the liver transcriptome data of 206 patients of NAFLD with different degrees of liver fibrosis for analysis. First, the enrichment scores of the critical transcriptome signatures in each liver sample of GSE135251 were determined using GSVA, and then NMF was used to explore the molecular subsets of NAFLD. By performing NMF clustering analysis on these 206 liver tissue samples, we found four NAFLD molecular subsets (Fig. 3A and B), including subset 1 (Cluster 1, C1; shown in blue), subset 2 (Cluster 2, C2; shown in orange), subset 3 (Cluster 3, C3; shown in cyan) and subset 4 (Cluster 4, C4; shown in red) (Fig. 3C). In C1, the NAFL signature enrichment score was significantly higher than in patients with other types of NAFLD, and patients in C1 subset had predominantly hepatic steatosis with less fibrosis and inflammatory infiltration. The C2 subset had the most significant enrichment of mononuclear-macrophage transcriptomic signatures, and the enrichment score of liver aging signature was also significantly higher in C2 subset, so patients with C2 subset may have the most severe liver aging with significant mononuclear-macrophage accumulation in liver tissues especially the Kupffer cells. The C3 subset was significantly enriched in non-myeloid immune cells such as CD4^+^ T cells, CD8^+^ T cells, NK cells, and B cells. Subset C4 was a predominantly liver fibrosis subtype, in which the enrichment scores for fibrosis transcriptomic signature, HSC-associated transcriptomic signatures and TGF-β signature were significantly higher than other NAFLD subsets, and patients in this subset had more advanced liver fibrosis or a higher risk of liver fibrosis progression than other NAFLD subsets (Fig. 3C). In the NMF clustering analysis of NAFLD liver samples from GSE135251, there were significant differences in the clinical characteristics across the four NAFLD molecular subsets such as NAS score and fibrosis grade in liver biopsies (Fig. 3C). Biopsies of patients' liver tissues showed that patients with advanced fibrosis were mostly clustered in the C4 subset, and NAFLD patients of C4 subset had significantly increased fibrosis scores compared with other subsets, and thus the NMF-based molecular typing of fibrosis in this study was consistent with the clinicopathological findings (Fig. 3D). Therefore, we found four molecular subtypes of NAFLD with significant differences in clinical characteristics, among which C4 subtype was the molecular subtype of fibrosis in NAFLD, and the patients with this subtype have more advanced liver fibrosis or may have a higher risk of liver fibrosis progression.

**Fig. 4:**
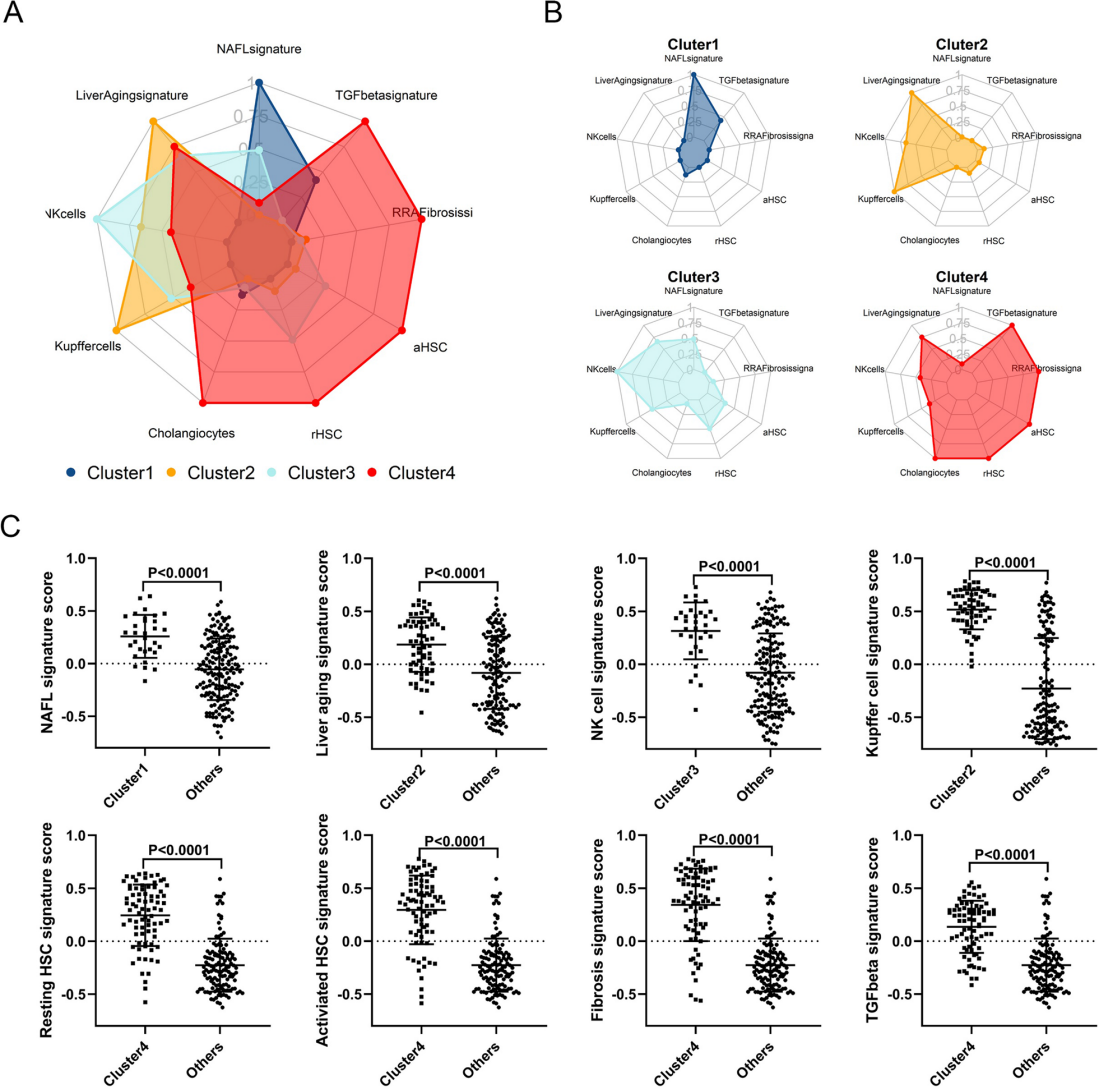
Comparison of the molecular features among different clusters. **A** Radar chart showing the difference in the molecular features across those 4 clusters. **B** Radar chart showing the characteristics of molecular features in each cluster. **C** Differences in the key molecular features across those clusters

After completing the molecular subtyping, we analyzed the differences in key transcriptomic signatures across molecular subsets. As shown in Fig. 4, compared with other subsets, the C4 fibrosis molecular subset showed significant fibrotic features, with significantly higher enrichment scores for key transcriptomic signatures such as fibrosis signature, TGF-β signature, and aHSC signature than other subsets (Fig. 4A–C). Enrichment score for the transcriptomic signature of choanocytes for C4 fibrosis molecular subset were also significantly higher than the other subsets, suggesting that choanocytes may play a critical role in the progression of fibrosis in NAFLD patients (Fig. 4A and B). In addition, the liver aging signature was also enriched in C4 fibrosis molecular subset, suggesting that liver aging was also an important factor that could exacerbate the progression of liver fibrosis in NAFLD patients (Fig. 4A and B). We found that compared with other subsets, the C4 fibrosis molecular subset showed obvious fibrosis characteristics, and the severity of liver fibrosis was substantially correlated with liver aging.

**Fig. 5:**
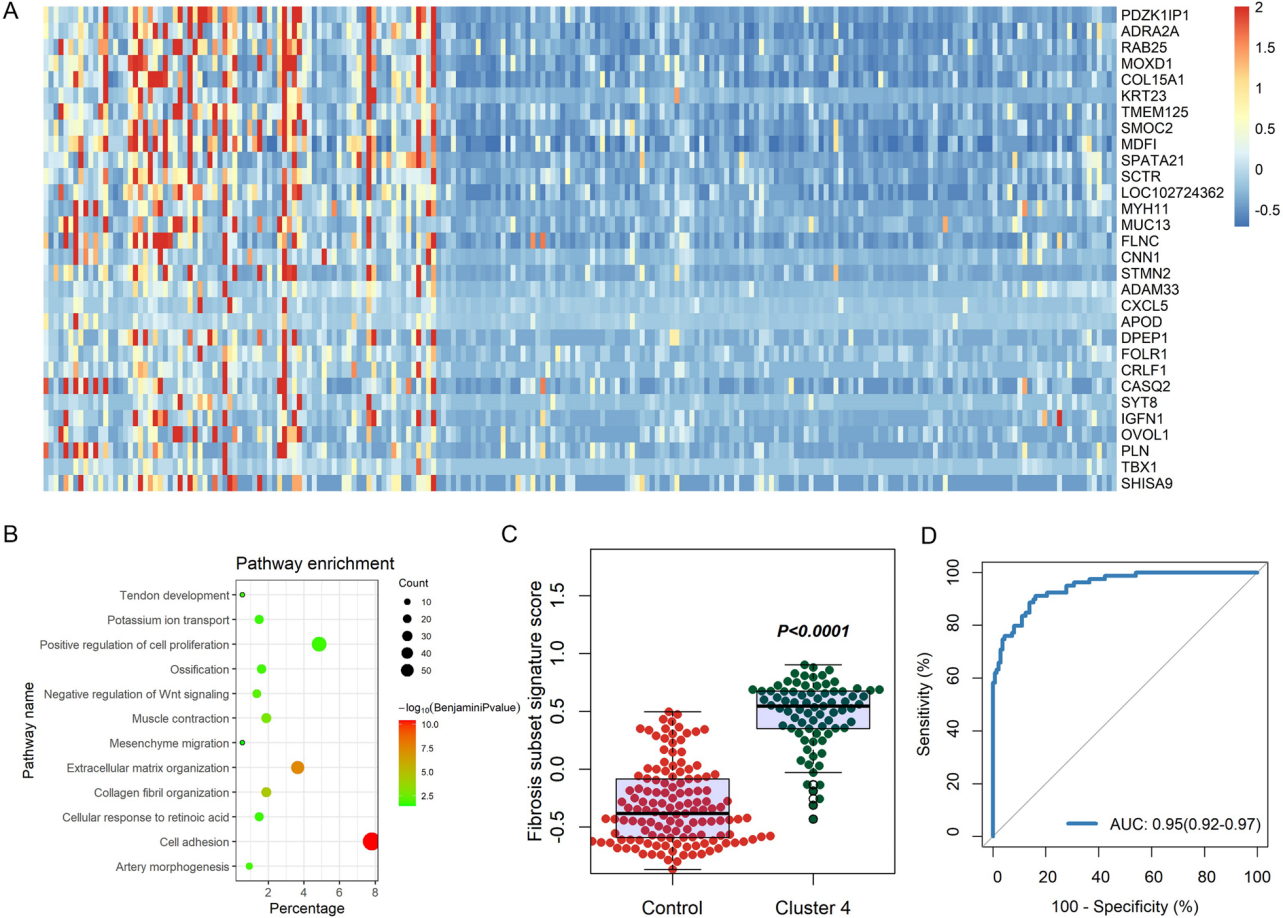
Development and validation of the transcriptomic signature for fibrosis subset of NAFLD. **A** Heatmap shows the main differentially expressed genes (DEGs) in the fibrosis subset compared to other subsets. **B** The functional GO pathways enriched in DEGs in the fibrosis subset. **C** The GSVA enrichment score for the fibrosis subset signature was significantly higher than that in other subsets. **D** ROC analysis shows that the GSVA enrichment score for the fibrosis subset signature can efficiently classify the fibrosis subset from other types

We further analyzed the signature genes that were significantly differentially expressed between C4 subset and other subsets, and found that the DEGs in C4 subset were mainly enriched in signaling pathways such as collagen and cell adhesion by GO functional enrichment pathways (Fig. 5A and B). We then constructed a transcriptomic signature for this fibrosis subset (Fibrosis subset signature) based on genes highly expressed in C4 subset compared with other subsets. The GSVA enrichment score of fibrosis subset signature was significantly different between C4 subset and other subsets, and could effectively identify liver tissues belonging to C4 subset (Fig. 5C and D). Therefore, we successfully established a transcriptomic signature that can effectively identify C4 fibrosis molecular subset.

In this subsection, we analyzed NAFLD molecular typing by NMF method, and found that there are four NAFLD molecular typing with significant differences in clinical characteristics, among which Cluster 4 subtype is closely related to liver fibrosis, and the severity of fibrosis in patients with this molecular subtype is more significant, and it is significantly related to liver aging. In addition, we also defined a transcriptomic signature that can effectively identify C4 fibrosis subtypes.

### Assessment the relationship between profibrotic macrophages and the fibrosis subset of NAFLD

In the development of NASH and fibrosis, our prior research revealed a critical role for immunological inflammation [11]. In addition, several studies have shown that different macrophage subsets have different roles in liver fibrosis and that certain macrophage subsets may be closely associated with liver fibrosis progression [39–44]. However, no study had explored the roles and the changes of those common macrophage subsets in the fibrosis subset of NAFLD, and the correlation between these macrophage subsets and the severity of NAFLD fibrosis was still unclear. In this study, we established macrophage subset-specific transcriptomic signatures based on liver scRNA-seq data that could be used to assess the number of monocyte-macrophage subsets in the liver and help to analyze the changes of each cell subset in the molecular subsets of NAFLD and fibrosis progression.

**Fig. 6:**
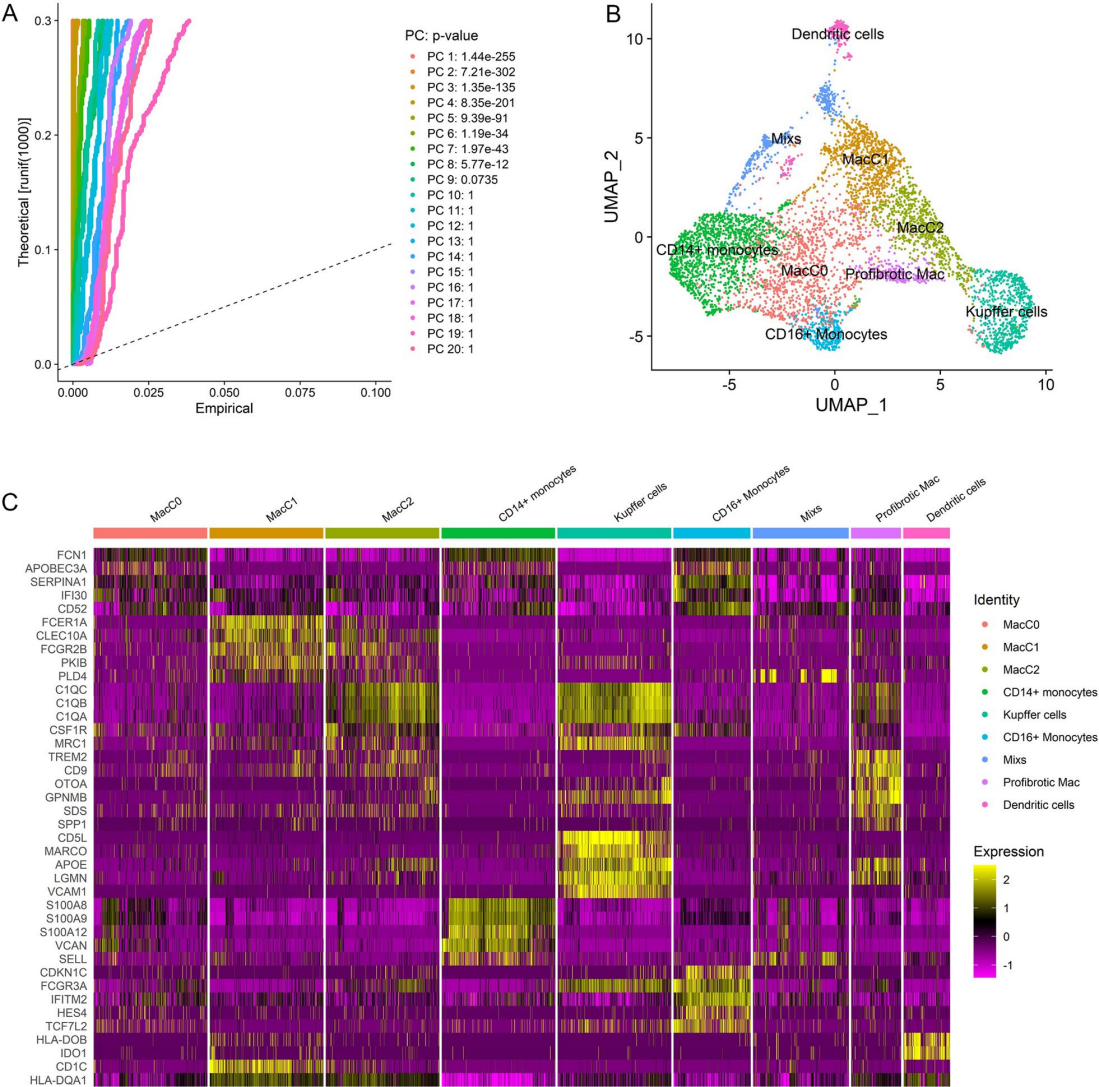
Identification of signature gene sets for liver monocyte-macrophage subsets via scRNA-seq analyses. **A** Determination of the statistical significance of PCA scores with JackStraw method. **B** UMAP plot of monocyte-macrophage subsets in the liver tissues. **C** Heatmap showing the expression of key genes in different monocyte-macrophage subsets of liver tissues

**Fig. 7:**
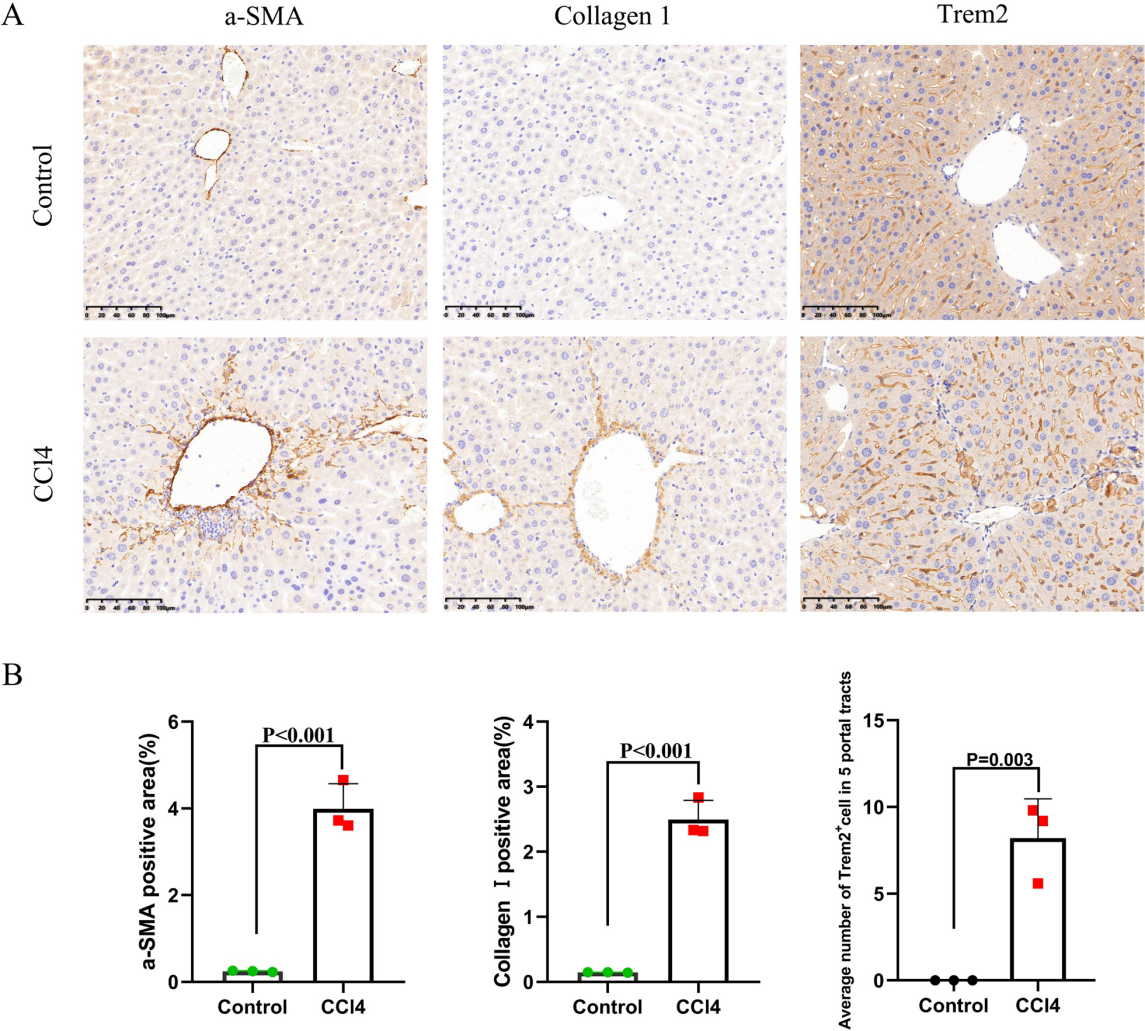
Expression of Trem2 was significantly increased in areas of advanced fibrosis in liver tissue of CCl4-induced DBA/2J mice. **A** Immunohistochemical staining images of α-SMA, Collagen 1 and Trem2 (magnification ×200); **B** Positive immunoreactivity analysis of α-SMA, Collagen 1 and Trem2 in liver sections (magnification × 200)

**Fig. 8:**
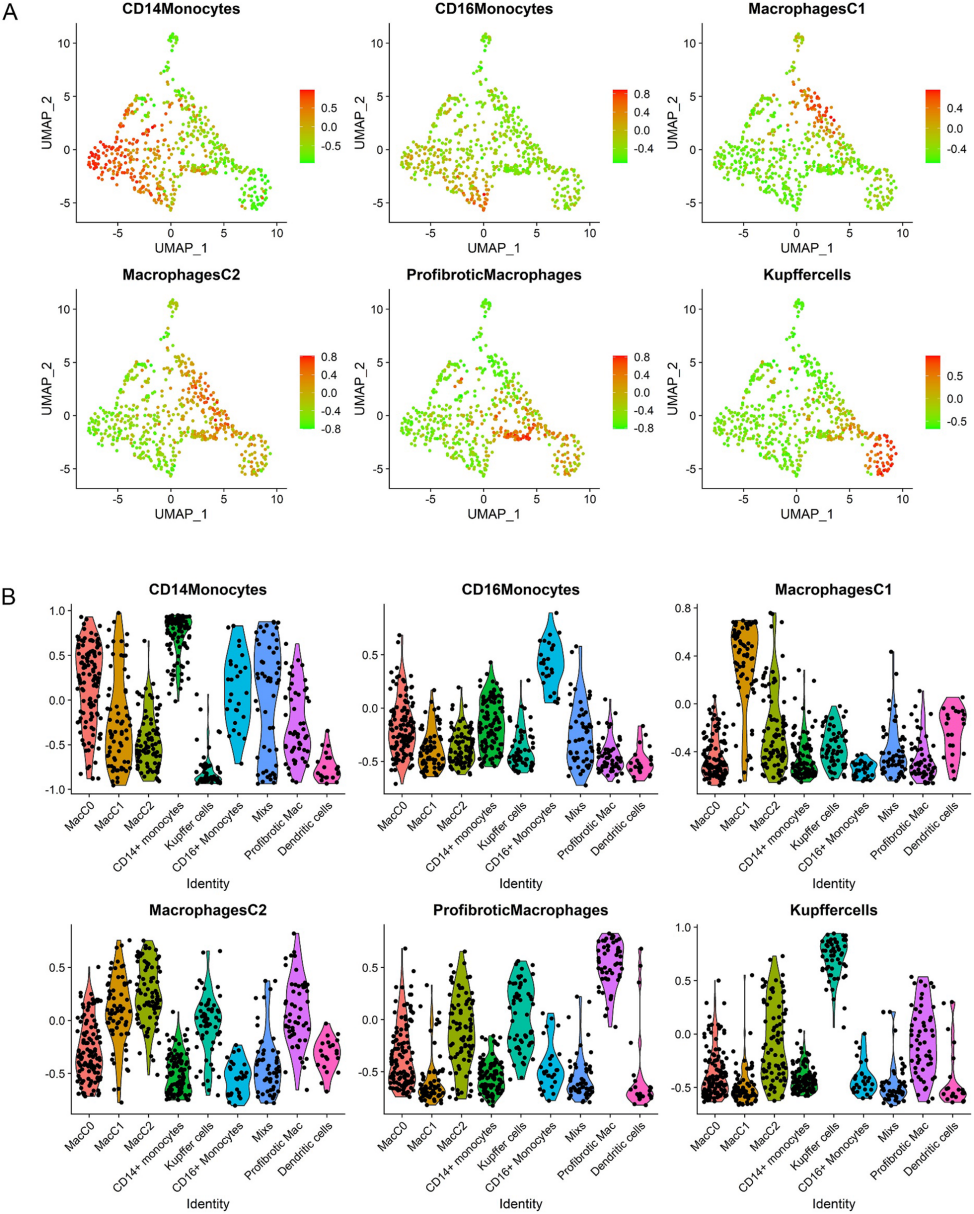
Establishment and validation of transcriptomic signatures for the monocyte-macrophage subsets of the liver by scRNA-seq analyses. **A** GSVA revealed that the subset-specific signatures were obviously enriched in each monocyte-macrophage subset. **B** Comparison of the difference in the enrichment scores of the subset-specific signatures in each monocyte-macrophage subset of the liver by violin plots

To establish the transcriptomic signatures of liver monocyte-macrophage subsets, we analyzed scRNA-seq data from CD45^+^ cells using the GSE136103 dataset for analysis. We included 9 liver samples with good quality single-cell transcriptomes, which were GSM4041150, GSM4041155, GSM4041158, GSM4041160, GSM4041161, GSM4041164, GSM4041166, GSM4041168 and GSM4041169. In addition, we included monocyte-macrophages of non-cancer liver from GSE140228 dataset. Before performing scRNA-seq analysis, we isolated monocyte-macrophages from the above samples and subsequently integrated them by the SCTransform method. By using the UMAP algorithm, these cells were divided into 9 clusters, including Kupffer cells, profibrotic macrophages, CD14^+^monocytes, CD16^+^monocytes and et.al (Fig. 6A and B). We further identified the signature genes expressed in each cell subsets by scRNA-seq analysis and constructed transcriptomic signatures for key cell populations (Fig. 6C, Additional file 2: Table S3). We selected the top 10 or top 20 genes highly expressed in specific cells to establish transcriptomic signatures that could represent the corresponding cell subsets (Fig. 6C, Additional file 2: Table S3). We found that genes such as CD5L, MARCO and TIMD4 were significantly highly expressed in Kuffer cells, and molecules such as CD9, TREM2 and SPP1 were significantly highly expressed in profibrotic macrophages, which was consistent with previous studies and proved the appropriate clustering in this study (Additional file 1: Fig. S5). The macrophage C2 (MacC2) subset highly expressed molecules such as MRC1 (CD206) and TREM2, which were similar to the immune phenotype of conventional M2 macrophages. In addition, using CCl4-induced DBA/2J mouse liver fibrosis model, we found that expression of Trem2 was significantly increased in mice with advanced liver fibrosis (Fig. 7A and B, Additional file 1: Fig. S6). We assessed the enrichment of these transcriptomic signatures in one liver sample (GSM4041169) by GSVA in order to determine if the aforementioned transcriptomic signatures could be utilized to characterize the corresponding cell subsets (Fig. 8A and B). The results showed that each transcriptomic signature was significantly enriched in the respective cell subset, indicating that those transcriptomic signatures might accurately reflect the appropriate cell subsets (Fig. 8A and B). Therefore, we established transcriptomic signatures that can represent the corresponding cell subsets through scRNA-seq analysis, including liver monocyte-macrophage subsets.

**Fig. 9:**
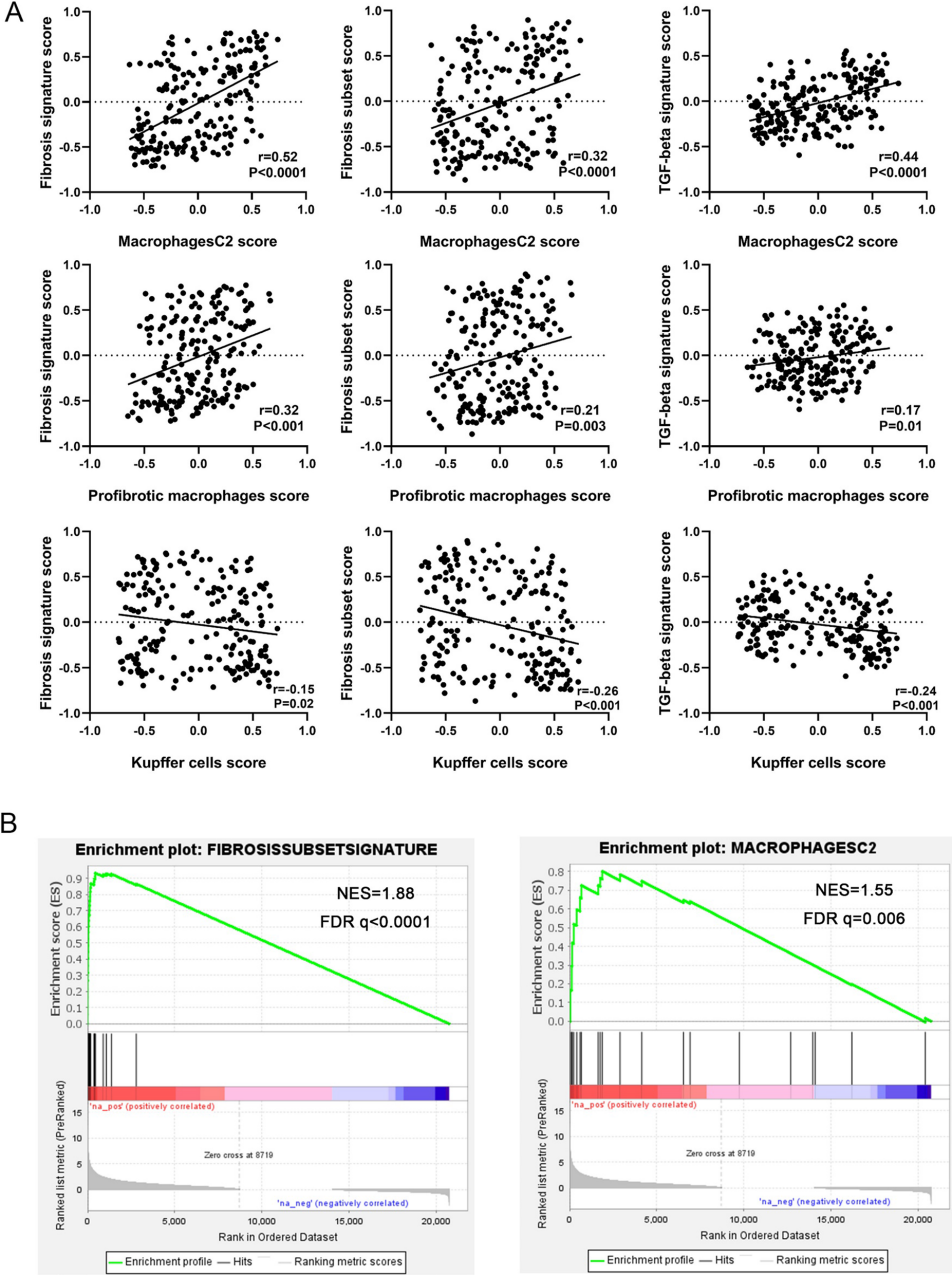
Correlation analyses of key monocyte-macrophage subsets with liver fibrosis in NAFLD patients. **A** Correlation of key monocyte-macrophage subsets with liver fibrosis markers by linear correlation analyses. **B** GSEA analyses showed the significantly increased enrichment of Fibrosis subset signature (left) and Macrophage C2 signature (right) in the liver of NAFLD patients with severe fibrosis

To clarify the roles of monocyte-macrophage subsets in the progression of liver fibrosis or the fibrosis subset in NAFLD, we first explored the correlations between those monocyte-macrophage subsets and liver fibrosis signatures by linear correlation analysis in the GSE135251 dataset. According to the findings, the GSVA enrichment score of fibrosis signature, fibrosis subset, and TGF-beta signature were all substantially correlated with MacC2 transcriptome signature (r = 0.52, P < 0.0001; r = 0.32, P < 0.0001; r = 0.44, P < 0.0001). The GSVA enrichment score of profibrotic macrophages signature were also significantly correlated with fibrosis signature score, fibrosis subset score and TGF-beta signature score (r = 0.32, P < 0.001; r = 0.21, P = 0.003; r = 0.17, P = 0.01). The GSVA enrichment score for the Kupffer cell transcriptomic signature was negatively correlated with the fibrosis signature score, fibrosis subset score and TGF-beta signature score (r = -0.32, P = 0.02; r = − 0.26, P < 0.001; r = -0.24, P < 0.001) (Fig. 9A). In addition, we also identified the significant enrichment of fibrosis subset signature and MacC2 signature in patients with advanced liver fibrosis by GSEA (NES = 1.88, FDR q < 0.0001; NES = 1.55, FDR q = 0.006) (Fig. 9B). The above results indicated that the profibrotic macrophages and MacC2 subset (similar to conventional M2-type macrophages) were both significantly correlated with the progression of liver fibrosis or the fibrosis subset in NAFLD patients.

We further explored the associations of key monocyte-macrophage subsets with fibrosis of liver in patients with Cluster 4 subset (Fibrosis molecular subset) (Additional file 1: Fig. S7). Both MacC2 score and the profibrotic macrophages score were significantly and positively correlated with fibrosis signature score, fibrosis subset score and TGF-beta signature score, demonstrating that these two macrophage subsets were closely associated with fibrosis progression in the fibrosis molecular subset of NAFLD (Additional file 1: Fig. S7). In contrast, Kupffer cells score in patients with cluster 4 subset was not significantly correlated with fibrosis signature score, fibrosis subset score and TGF-beta signature score (P > 0.05), suggesting that Kupffer cells did not have a significant role in the progression of fibrosis in the fibrosis molecular subset of NAFLD (Additional file 1: Fig. S7).

The above results in this subsection indicated that the profibrotic macrophages and MacC2 subset were both significantly associated with the progression of liver fibrosis in patients of NAFLD with Cluster 4 subset.

## Discussion

At present, there are huge challenges in the diagnosis and treatment of NAFLD patients combined with fibrosis, which are partially caused by our limited understanding of liver fibrosis in NAFLD [3, 8, 45]. Therefore, it is necessary to further explore the pathogenic factors and related mechanisms of liver fibrosis in NAFLD. Through accurately defining the molecular subtypes related to fibrosis progression and the underlying pathogenic factors, subtype-based personalized therapy for NAFLD with fibrosis or at high risk of liver fibrosis progression may be possible in the future.

Although we currently have a clear consensus on the clinical heterogeneity of fibrosis severity that exists in NAFLD patients, a precise understanding of this heterogeneity at molecular level is lacking. In this study, we analyzed the molecular subsets of NAFLD by NMF and found that there are four main subsets of NAFLD including C1, C2, C3 and C4. C1 subset is mainly characterized by hepatic steatosis, and patients with C1 subset have no or modest liver fibrosis and inflammatory infiltrates. C2 subset is mainly characterized by increased accumulation of mononuclear-macrophages especially Kupffer cells in the liver, and patients of this subset generally have serious liver aging. C3 subset is mainly characterized by increased accumulation of non-myeloid immune cells such as CD4^+^ T cells, CD8^+^ T cells, NK cells, and B cells. The last subset is C4, which is mainly characterized by liver fibrosis. Patients with C4 subset have more advanced liver fibrosis than patients with other subsets, or may have a high risk of liver fibrosis progression.

Multiple omics studies have shown that the gene expression profile of liver tissues can change with the progression of NAFLD, for example, the expression levels of lipid metabolism-related genes are significantly increased after hepatic fat deposition and steatosis, and the expression levels of lipid metabolism-related genes are significantly increased after NASH progression or liver fibrosis [46–49]. In the progressive stage of NAFLD, the expression levels of genes related to inflammation and fibrosis also increase significantly, so there is a significant correlation between the expression levels of some specific genes in liver tissues and the severity of NAFLD disease activity and clinical progression stage [46–49]. The detection of the expression levels of these genes may thus help to define the clinical subtype or disease severity of NAFLD, or help to predict the risk of disease progression [50, 51]. The findings from the transcriptomic analyses in our study suggested the distinct molecular subsets of NAFLD, and found a novel fibrosis subset which had more advanced liver fibrosis than other subsets. Therefore, through detecting the expression levels of key genes, the molecular subtypes including the fibrosis subtype of NAFLD may be accurately defined, and personalized precision therapy by molecular subtype may be achieved. However, more future studies are necessary to expand our knowledge on this aspect.

Pathological tissue repair in the liver tissues leads to hepatic fibrosis, which is characterized by an excessive synthesis and deposition of extracellular matrix (ECM) [52]. In liver fibrosis, activated HSCs are the primary source of ECM [53]. HSCs are in a resting state under physiological settings and maintain ECM homeostasis [53]. However, when under pathogenic conditions of promoting fibrosis progression, pro-fibrotic factors produced by inflammatory cells and other factors can activate HSCs and transform them into myofibroblast-like cells, resulting in the excessive synthesis of collagen fibers and the formation of large amounts of fibrous tissues [53]. Multiple factors, including TGF-β and platelet-derived growth factor (PDGF), are involved in the stimulation of HSCs [54, 55]. At present, the mechanism of HSCs over-activation in the fibrosis progression of NAFLD is not fully understood and need to be explored.

By analyzing transcriptomic data from liver tissues of NAFLD patients combined with fibrosis, our previous study found that, in addition to ECM production-related pathways, immune-related pathways also play an important role in the progression of fibrosis in NAFLD [11]. In addition, emerging studies have shown that myeloid immune cells in the liver such as monocyte-derived macrophages are key immune cells involved in liver fibrosis [41, 42, 56, 57]. Liver macrophages mainly include resident Kupffer cells and macrophages differentiated from circulating monocytes [40, 58]. Macrophages exhibit obvious heterogeneity in different subsets and have strong plasticity under different physiological and pathological processes [56]. Macrophages are routinely classified as pro-inflammatory macrophages (M1) and anti-inflammatory macrophages (M2) [56], and some studies have found that M2-type macrophages correlate with the severity of liver fibrosis [59]. Macrophages in the liver can be involved in the progression of liver fibrosis by secreting TGF-β to activate HSC [57], but can also play a key role in the regression of fibrosis by promoting extracellular matrix degradation [60].

In the present study, we further explored the roles of different liver myeloid immune cell subsets in liver fibrosis progression of NAFLD patients via comprehensive analyses of the molecular subsets of NAFLD. We found that MacC2 and profibrotic macrophages were both significantly associated with the enrichment score of fibrosis subset in patients with NAFLD (Fig. 9). Compared with other monocyte-derived macrophages, the MacC2 subset is characterized by high expression of MRC1 (CD206) and is similar to the immune phenotype of conventional M2 macrophages. Two recent studies reveal that a novel profibrotic macrophage subset differentiated from circulating monocytes, CD9^+^TREM2^+^ macrophage, is significantly increased in patients with liver cirrhosis and plays a key role in activating HSCs at least via releasing TGF-β [61, 62]. The results of this study showed a significant correlation between this profibrotic macrophage subset (CD9^+^TREM2^+^ macrophages) and the severity of liver fibrosis in patients with NAFLD. Those findings above suggest that M2 macrophages and the profibrotic macrophage subset are both involved in the liver fibrosis progression of NAFLD patients.

The molecular mechanisms underlying the progression of liver fibrosis in NAFLD patients are far from clear [15]. The progression of liver fibrosis in NASH patients is still not effectively treated, thus more investigation into the primary molecular processes of NASH liver fibrosis and identification of important therapeutic targets is required [8]. Clarifying the molecular mechanism of liver fibrosis progression will help to find effective targets for intervention therapy [63]. Our study revealed the fibrosis molecular subset of NAFLD, which had more advanced liver fibrosis than other subsets or may have a high risk of fibrosis progression. Moreover, the fibrosis subset was significantly correlated with the profibrotic macrophage subset. This finding suggests that the profibrotic macrophage subset may be an important player in the progression of liver fibrosis of NAFLD patients. Further studies are needed to uncover the possible mechanisms underlying the role of profibrotic macrophages in the progression of liver fibrosis of NAFLD patients, and to explore whether this macrophage subset is a promising treatment target to prevent or reverse the progression of liver fibrosis.

The only approach currently used to reliably distinguish NASH from NAFLD and precisely gauge the degree of fibrosis is liver biopsy, although this method has limitations related to sampling error and pathologist experience that may compromise the accuracy and completeness of the diagnosis [64, 65]. For instance, the definitions of NASH are not completely consistent among different pathologists. A study found that 20.6% of 247 patients with NASH who were enrolled on the basis of initial liver biopsy did not actually have NASH-related pathological changes [66]. Therefore, more accurate diagnostic methods in assessing the liver fibrosis in NAFLD need to be developed. In this study, we developed a fibrosis subset transcriptomic signature which was significantly enriched in patients with the C4 subset (Fibrosis subset) and other subsets and could effectively identify liver tissues belonging to C4 subset. Transcriptomic assessment via the fibrosis subset signature may help to determine the fibrosis severity status in liver tissue or predict whether the patient is at high risk of fibrosis progression.

Our study revealed the molecular subsets of NAFLD by integrating key information from transcriptomic expression profiling and liver microenvironment, and identified a novel and distinct fibrosis subset of NAFLD. The fibrosis subset is significantly different from other NAFLD subtypes, and is significantly correlated with the profibrotic macrophages and M2 macrophage subset. These two liver macrophage subsets may be important players in the progression of liver fibrosis of NAFLD patients and are promising therapeutic targets for inhibiting fibrosis progression. More future studies are needed to uncover both the clinical features and the underlying mechanisms of this fibrosis subset in NAFLD.

## Supplementary Information

**Additional file 1: Figure S1.** Integration analyses of transcriptomic data of NAFL patients and healthy controls (A, Heatmap in the RRA analyses of 6 liver transcriptome datasets comparing NAFL with controls. The number was for the log2 value of fold change. Red color indicated the up-regulation of gene expression in the NAFL liver tissues, while green color indicated the down-regulation of gene expression in the NAFL liver tissues. B, Comparison the difference in the GSVA enrichment score of NAFL transcriptomic signature between NAFL patients and controls. C, Assessment of the diagnostic role of NAFL transcriptomic signature through ROC method). **Figure S2.** Development of liver aging-related transcriptomic signature (A, Heatmap in the RRA analyses of 4 liver transcriptome datasets comparing older individuals with younger individuals. B, Comparison the difference in the GSVA enrichment score of liver aging transcriptomic signature between older individuals and younger individuals. C, Significant linear correlation between liver aging signature score and real age). **Figure S3.** Development of HSCs activation signature via transcriptomic analyses of TGF-β stimulated HSCs in GSE148849 (A, Heatmap in the transcriptomic analyses of GSE148849 comparing TGF-β-stimulated HSCs with non-stimulated HSCs. B, Volcano map of differentially expressed genes in the transcriptomic analyses. C, Comparison of the difference in the GSVA enrichment score of HSCs activation signature between TGF-β-stimulated HSCs and non-stimulated HSCs). **Figure S4.** Analyses of liver single cell transcriptomic data in GSE174748 (A, t-SNE plot of cell clusters in liver tissues; B, UMAP plot of cell clusters in liver tissues; C, Heatmap showing the expression of key genes in different cell clusters of liver tissues). **Figure S5.** Expression of some feature genes in the monocyte-macrophage subsets of the liver. **Figure S6.** Examination of histopathological damage and fibrosis levels in the liver by H&E and Masson staining. (A. Representative image of H&E and Masson-stained liver sections (magnification × 200); B. Statistics of Masson positive area (%)). **Figure S7.** Correlation analyses of key monocyte-macrophage subsets with liver fibrosis in NAFLD patients of Cluster 4 (Fibrosis subset).

**Additional file 2: Table S1.** Gene lists of the transcriptomic signatures developed in this study. **Table S2.** Characteristics of liver transcriptome datasets in this study. **Table S3.** Gene lists of the transcriptomic signatures developed for monocyte-macrophage subsets in liver.

## Data Availability

The public datasets were downloaded and analyzed in this study, which can be found in GEO data repository.

## Abbreviations

NAFLD: Non-alcoholic fatty liver disease
T2DM: Type 2 diabetes mellitus
NASH: Non-alcoholic steatohepatitis
NAFL: Non-alcoholic fatty liver
ECM: Extracellular matrix
HSCs: Hepatic stellate cells
TGF-β: Transforming growth factor beta
PDGF: Platelet-derived growth factor
NMF: Non-negative matrix factorization
GEO: Gene Expression Omnibus
scRNA-seq: Single-cell RNA sequencing
RNA-seq: RNA-sequencing
RRA: Robust rank aggregation
DEGs: Differentially expressed genes
Log2FC: Log2 fold change
GSVA: Gene set variation analysis
GSEA: Gene set enrichment analysis
NES: Normalized enrichment score
FDR: False discovery rate
ROC: Receiver Operating Characteristic
