# Supplementary material for: Identifying a distinct fibrosis subset of NAFLD via molecular profiling and the involvement of profibrotic macrophages
Source: J Transl Med. 2023 Jul 6;21:448. doi: 10.1186/s12967-023-04300-6 (PMC10326954; doi:10.1186/s12967-023-04300-6)
Supplement: Supplementary file 1 — Additional file 1: Figure S1. Integration analyses of transcriptomic data of NAFL patients and healthy controls (A, Heatmap in the RRA analyses of 6 liver transcriptome datasets comparing NAFL with controls. The number was for the log2 value of fold change. Red color indicated the up-regulation of gene expression in the NAFL liver tissues, while green color indicated the down-regulation of gene expression in the NAFL liver tissues. B, Comparison the difference in the GSVA enrichment score of NAFL transcriptomic signature between NAFL patients and controls. C, Assessment of the diagnostic role of NAFL transcriptomic signature through ROC method). Figure S2. Development of liver aging-related transcriptomic signature (A, Heatmap in the RRA analyses of 4 liver transcriptome datasets comparing older individuals with younger individuals. B, Comparison the difference in the GSVA enrichment score of liver aging transcriptomic signature between older individuals and younger individuals. C, Significant linear correlation between liver aging signature score and real age). Figure S3. Development of HSCs activation signature via transcriptomic analyses of TGF-β stimulated HSCs in GSE148849 (A, Heatmap in the transcriptomic analyses of GSE148849 comparing TGF-β-stimulated HSCs with non-stimulated HSCs. B, Volcano map of differentially expressed genes in the transcriptomic analyses. C, Comparison of the difference in the GSVA enrichment score of HSCs activation signature between TGF-β-stimulated HSCs and non-stimulated HSCs). Figure S4. Analyses of liver single cell transcriptomic data in GSE174748 (A, t-SNE plot of cell clusters in liver tissues; B, UMAP plot of cell clusters in liver tissues; C, Heatmap showing the expression of key genes in different cell clusters of liver tissues). Figure S5. Expression of some feature genes in the monocyte-macrophage subsets of the liver. Figure S6. Examination of histopathological damage and fibrosis levels in the liver by H&E and Masson st [file 12967_2023_4300_MOESM1_ESM.pptx]

## Slide 1
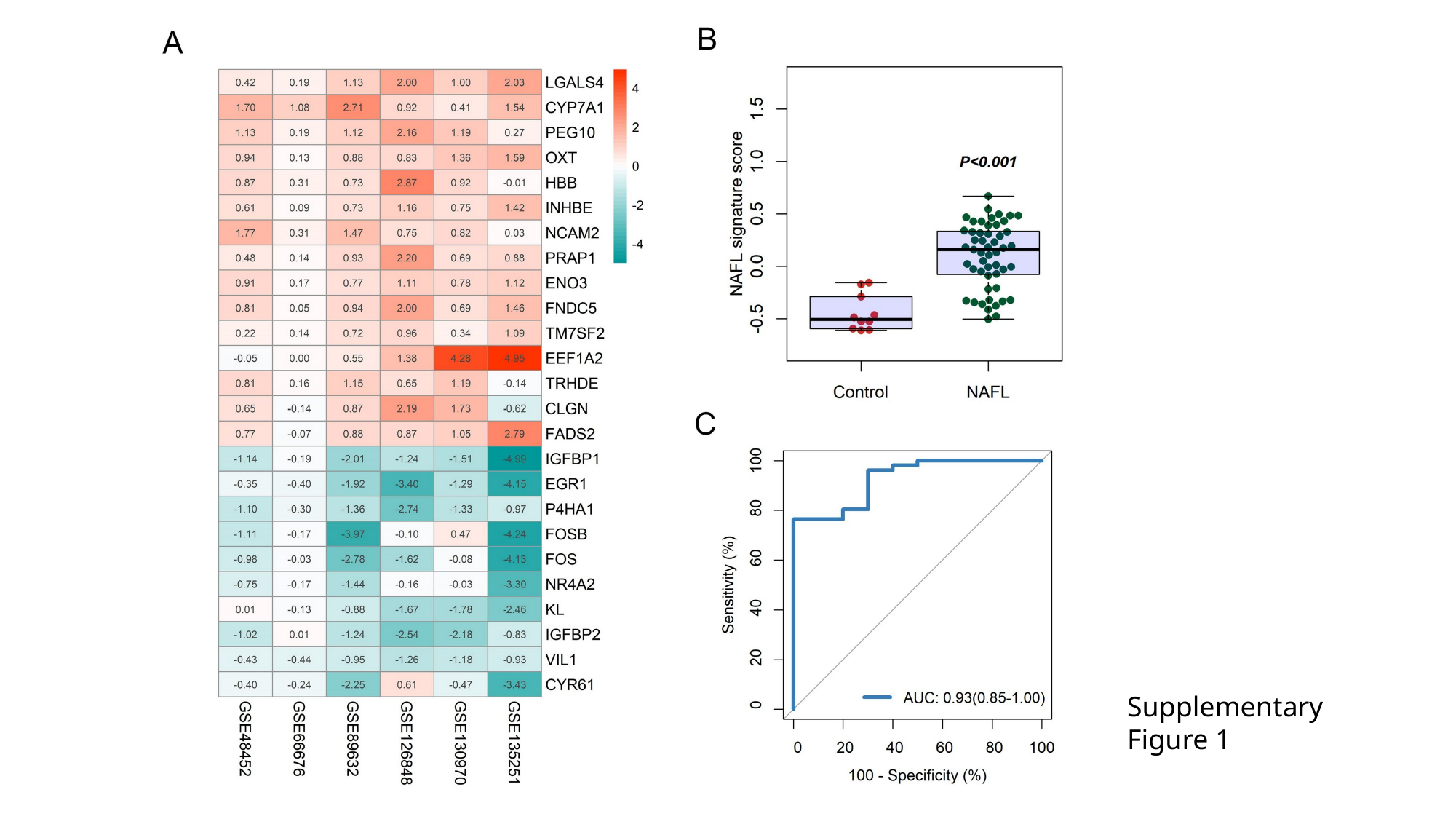

Supplementary Figure 1

## Slide 2
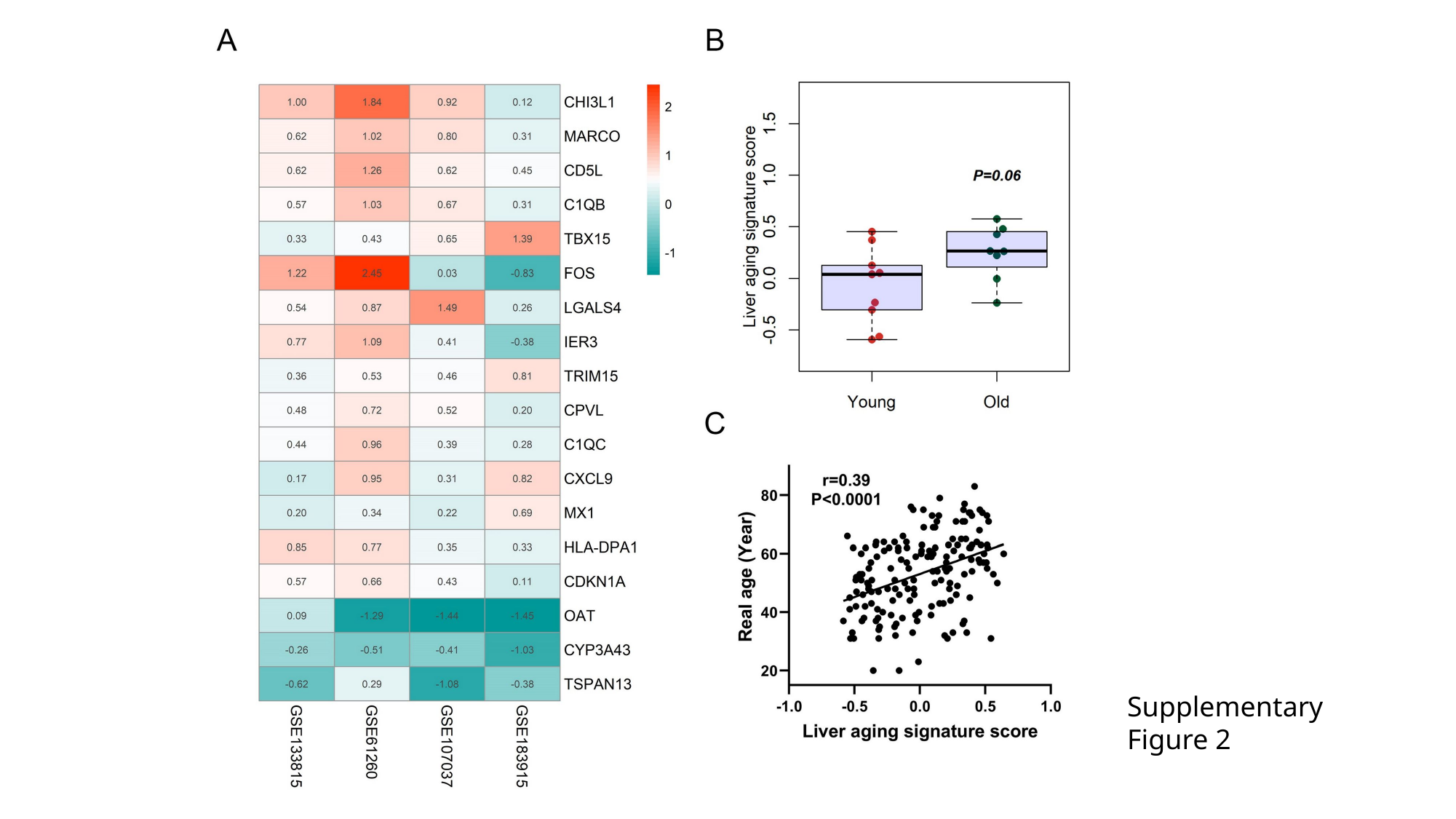

Supplementary Figure 2

## Slide 3
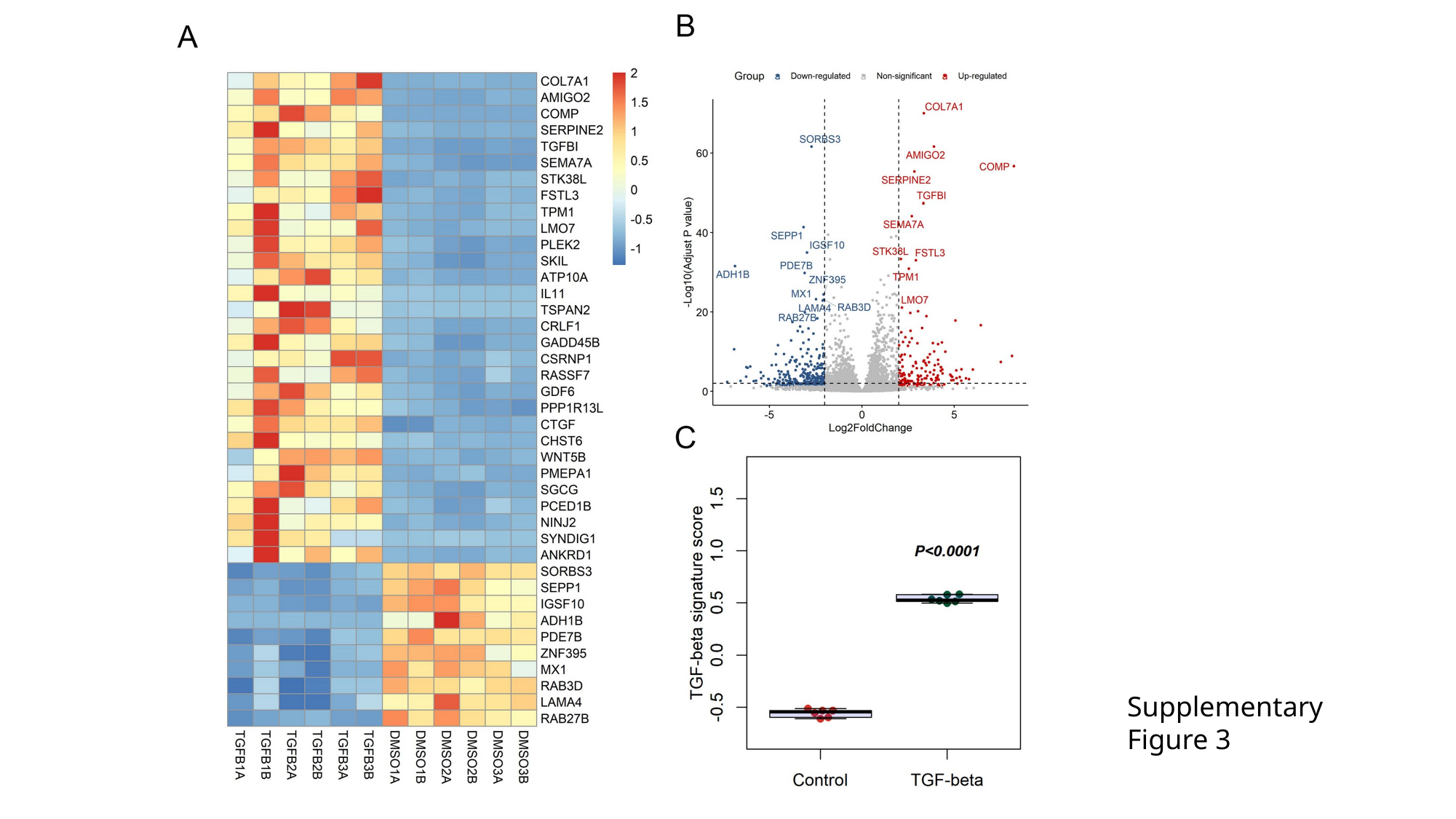

Supplementary Figure 3

## Slide 4
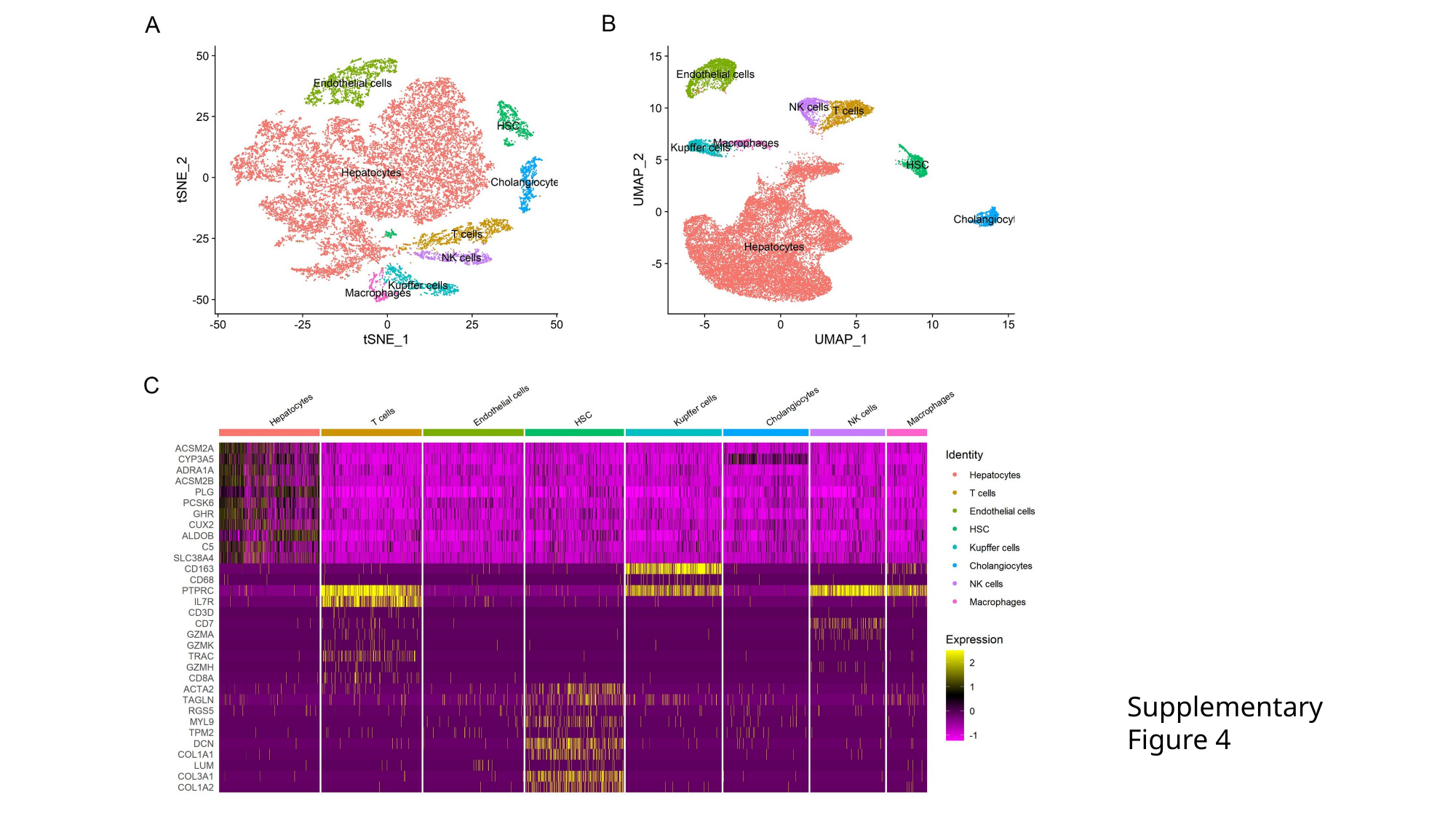

Supplementary Figure 4

## Slide 5
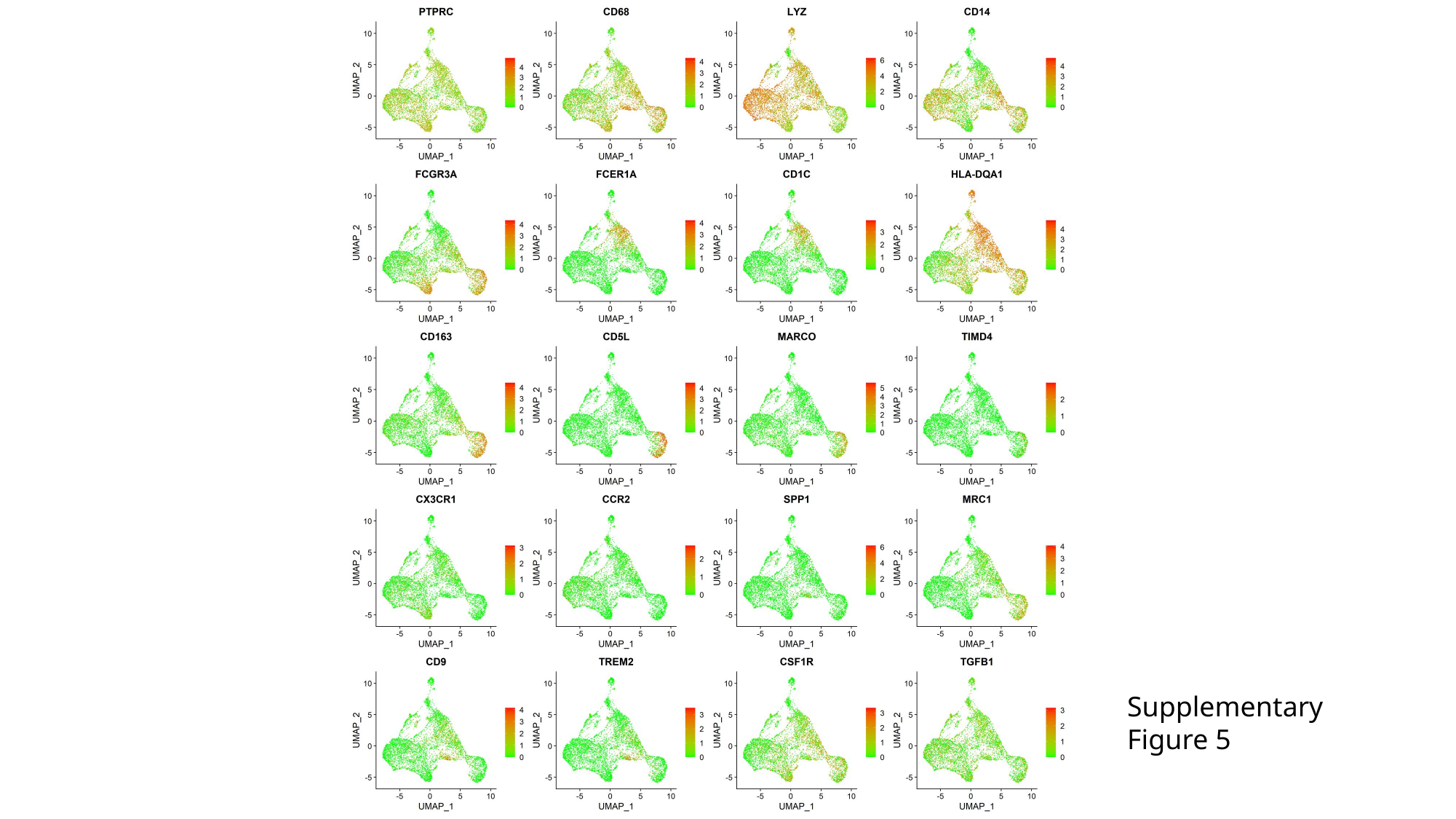

Supplementary Figure 5

## Slide 6
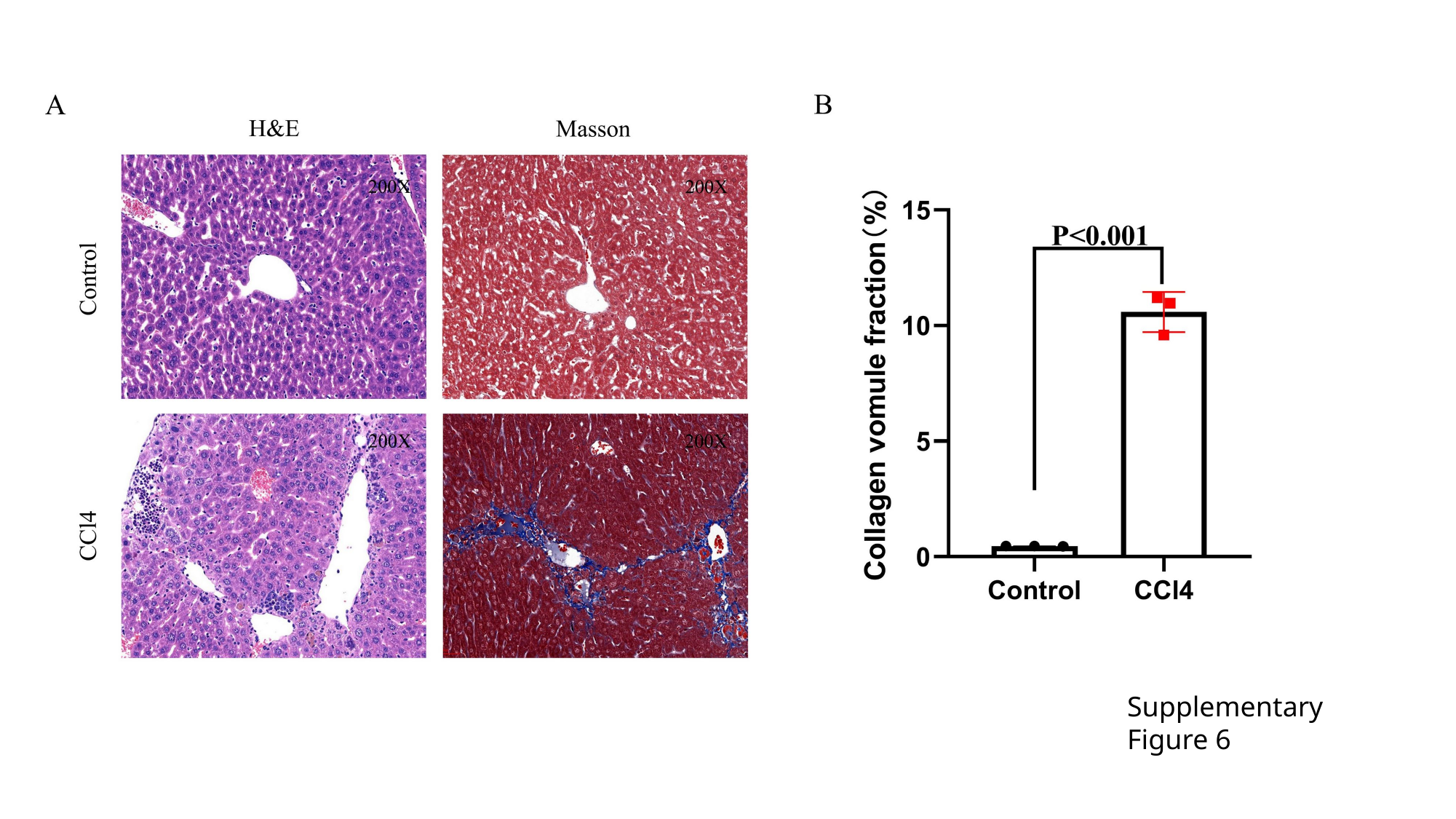

Supplementary Figure 6

## Slide 7
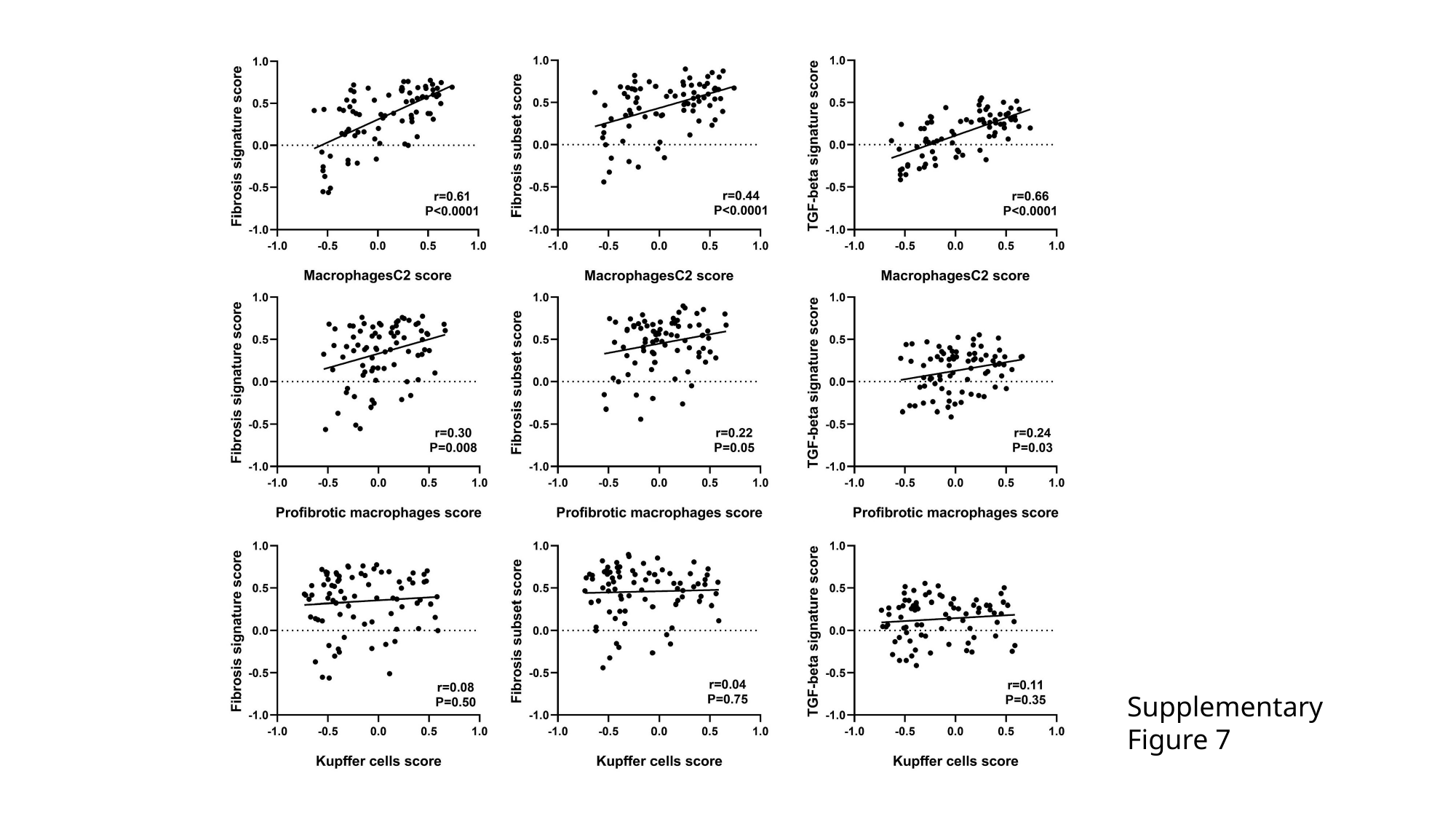

Supplementary Figure 7
