# Supplementary material for: Identifying a distinct fibrosis subset of NAFLD via molecular profiling and the involvement of profibrotic macrophages
Source: J Transl Med. 2023 Jul 6;21:448. doi: 10.1186/s12967-023-04300-6 (PMC10326954; doi:10.1186/s12967-023-04300-6)
Supplement: Supplementary file 2 — Additional file 2: Table S1. Gene lists of the transcriptomic signatures developed in this study. Table S2. Characteristics of liver transcriptome datasets in this study. Table S3. Gene lists of the transcriptomic signatures developed for monocyte-macrophage subsets in liver. [file 12967_2023_4300_MOESM2_ESM.docx]

**Supplementary table 1 Gene lists of the transcriptomic signatures developed in this study**

| **Gene signatures** | **Gene list** |
| --- | --- |
| NASH signature | SPP1, FBLN5, CHI3L1, CCL20, CD24, FABP4, GPNMB, VCAN, EFEMP1, CXCL10, DPT, STMN2, MMP7, LUM, AKR1B10, THBS2, BICC1, FABP5, EPHA3, LYZ, CXCL6, DCDC2, TYMS, LPL, FAT1, UBD, ITGBL1, IL32, ANXA2, BIRC3, COL1A2, SUSD2, HKDC1, CTSK, ACSL4, C7, DCN, TNFRSF12A, ASPN, CDKN1A, KRT7, SPON1, COL1A1, MOXD1, GPC4, CHST9, THY1, RNASE6, LXN, ADAMTSL2, PODN, LAMA2 IGFBP7, PTGDS, CXCL9, CDH11, TSPAN8, RGS4, EPCAM, GZMK, THBS1, MFAP4, GSN, ANTXR1, FMO2, DTNA, GEM, PRAMEF10, CXCL8, TC2N, SVEP1, COL3A1, CYR61, LAMC3, CFTR, PLCXD3, F13A1, MGP, LGALS3, LOXL4, CXCR4, FAM169A, AEBP1, FAP, CAPG, PLA2G7, GPC3, FGFR2, COL4A1, ANKRD1, SMOC2, F3 CDH6, UPP2, IL7R, ADAM28, SRPX2, MAP1B, PDGFRA, OLR1, AQP1, ANXA13, TREM2, CCDC80, SLC1A3, ZMAT3, NFASC, SLIT2, MMP2, ANXA1, GPRIN3, HLA-DQA1, GLS, CLIP4, SERPINE2, EEF1A2 |
| Fibrosis signature | AKR1B10, AEBP1, STMN2, CCL19, EPCAM, DCDC2, ITGBL1, EFEMP1, PDZK1IP1, CCL21, PTGDS, IL32, LGALS3, THBS2, LOXL4 LUM, CCL20, COL1A1, CXCL6, CXCL10, CDH11, SPON1, FXYD2, LAMC3, DPT, F3, ANXA13, CD24, COL1A2, LGALS4, TNFRSF12A, FAP SOX9, EEF1A2, PLP2, AQP1, GPX2, UBD, SPP1, LTBP2, TYMS, THY1, PLCXD3, SMOC2, GPC3, FABP5, CHST4, MMP7, BICC1, PDGFD, GSN, VTCN1, PROCR, LBH, DTL, ANXA2, CHST9, C7, SLC44A3, CLDN7, PODN, BEX2, ZWINT, FAM3B, FCER1A, SPINT2, FBLN5, FABP4, CLIC6, EDA2R, FAT1, LAMA2, S100A6, HKDC1, FAM169A, TIMP1, SLC1A7, KDELR3 |
| NAFL signature | LGALS4 , CYP7A1, PEG10, OXT, HBB, INHBE, NCAM2, PRAP1, ENO3, FNDC5, TM7SF2, EEF1A2, TRHDE, CLGN, FADS2, GPAM, PPP1R3C, SLC39A7, EIF1AY, LRRC31, ISM1, FDFT1, GCK, NPY6R, DHRS7, MAMDC4, RPS4Y1, TOR3A, SQLE, AKR1B10 |
| LiverAgingsignature | CHI3L1, MARCO, CD5L, C1QB, TBX15, FOS, LGALS4, PZP, IER3, TRIM15, CPVL, C1QC, CXCL9, MX1, CDKN1A, MX2, C1QA, CXCL13, CDHR2, SERPINE1, IGKC, CFD, SNX10, IFI16, IGFBP1, FREM2 CXCL10, CD74, LAPTM5, CD83 |
| HSCTGFbetasignature | COL7A1, AMIGO2, COMP, SERPINE2, TGFBI, SEMA7A, KDELR3, PDLIM7, STK38L, FSTL3, TPM1, PDGFC, SRA1, TNFRSF12A, SEC13, DLC1, ITGBL1, BHLHE40, RCN3, SPARC, UBE2V2, SURF4, NDEL1, GMPPB, PLCB4, LMO7, LRRC59, PLEK2, SEC23A, SKIL |
| Hepatocytes | ACSM2A, CYP3A5, ADRA1A, ACSM2B, PLG, PCSK6, GHR, CUX2, ALDOB, C5, SLC38A4 C9, PPARGC1A, C4BPA, PCK1, KNG1, ABCB4, HGD, AGMO, CYP3A4, HRG, F5, AOX1, SLC39A14, SLC7A2, FABP1, CRP, BHMT, LBP, HPX |
| Cholangiocytes | DEFB1, TM4SF4, KRT8, AMBP, VTN, SPP1, GC, FXYD2, KRT7, KRT18, AGT, CLDN10, RBP4, CD24, KRT19, LGALS4, EPCAM, MMP7, TACSTD2, ELF3, CLDN3, SLPI, CA2, TNFRSF12A, CXCL1, CXCL6, AKR1C1, FGGY, FGG, SH3YL1 |
| B cells | CD79A, MS4A1, TCL1A, CD79B, VPREB3, BANK1, IGHD, SPIB, FCER2, CD22, FCRLA, TNFRSF13C HLA-DOB, FAM129C, KIAA0125, BLK, CD19, CCR7, FCMR, P2RX5, RALGPS2, HVCN1, ARHGAP24, SELL, BCL11A, ADAM28, GAPT, NCF1, CYB561A3, POU2F2 |
| CD4 Tcells | LTB, TRAC, IL7R, CD3D, TRBC2, CD2, CD3E, TRBC1, CD40LG, CD3G, ACAP1, LCK, SPOCK2, SIT1, LAT, RHOH, TRAF3IP3, TRAT1, GPR171, FLT3LG, CD27, MAL, CCR7, RCAN3, BCL11B, CD6, CLEC2D, GPR183, ARHGAP15, TBC1D10C |
| CD8 Tcells | GZMK, CD3D, TRAC, GZMH, CD8A, CD3E, CD2, TRGC2, CD8B, TRBC2, GZMA, CD3G, CTSW, LCK, CST7, GPR171, KLRG1, IFNG, ACAP1, GZMM, TRBC1, SH2D1A, LAG3, CXCR6, RHOH, GRAP2, PTPN7, LAT, PRF1, SIT1 |
| Endothelial cells | FCN3, CRHBP, SLC9A3R2, RAMP2, CLEC14A, CLDN5, SPARCL1, PLVAP, PLPP1, VWF CCL14, RNASE1, HSPG2, SPARC, NPDC1, PLPP3, TIMP3, EGFL7, PCAT19, SDPR, RBP7, RAMP3, EMCN, FLT1, IL33, LDB2, CAV1, SPRY1, TSPAN7, HYAL2 |
| Kupffer cells | C1QC, CD5L, MARCO, CD163, MS4A7, CFD, HMOX1, FOLR2, SDC3, FCGR3A, CETP, CD68 CPVL, MS4A4A, VCAM1, AXL, VSIG4, FGL2, CREG1, CFP, MPEG1, CCL3L3, LILRB5, CYBB, MSR1, VMO1, MRC1, FAM26F, IGSF6, GPNMB |
| Macrophages | FCER1A, CLEC10A, HLA-DMB, HLA-DQA2, CPVL, GPR183, RGS10, RNASE6, RGS1, IL1B, MNDA, JAML, SPI1, CD1C, CD83, IGSF6, CD68, LGALS2, PHACTR1, IFI30, PLD4, VSIG4, PLAUR, PKIB, LY86, CD1E, CAPG, CLEC7A, FCGR2A, CSF2RA |
| Monocytes | S100A9, S100A8, FCN1, CSTA, VCAN, MNDA, S100A12, IFI30, SPI1, LGALS2, CFD, CYBB STXBP2, PLAUR, IL1B, CLEC12A, C5AR1, FGL2, CLEC7A, BCL2A1, APOBEC3A, CFP, JAML, TNFSF13B, NCF2, HCK, FPR1, CD68, PILRA, FAM26F |
| NK cells | GNLY, PRF1, CD7, XCL1, GZMA, KLRD1, CST7, KLRB1, GZMB, XCL2, CTSW, CCL5, KLRF1, FGFBP2, CD247, TRDC, IL2RB, CLIC3, HOPX, CD160, PYHIN1, GZMM, SPON2, CD69, MATK, IFNG, STK17A, ALOX5AP, KLRC1, APOBEC3G |

**Supplementary table 2 Characteristics of liver transcriptome datasets in this study**

| **GSE ID** | **Participants** | **Tissues** | **Methods** | **Platform** | **References** |
| --- | --- | --- | --- | --- | --- |
| GSE48452 | 14 NAFL patients and 14 controls | Liver | Microarray | GPL11532 | Ahrens M, Ammerpohl O, von Schönfels W, Kolarova J et al. DNA methylation analysis in nonalcoholic fatty liver disease suggests distinct disease-specific and remodeling signatures after bariatric surgery. Cell Metab 2013 Aug 6;18(2):296-302. |
| GSE66676 | 26 NAFL patients and 34 controls | Liver | Microarray | GPL6244 | Xanthakos SA, Jenkins TM, Kleiner DE, Boyce TW et al. High Prevalence of Nonalcoholic Fatty Liver Disease in Adolescents Undergoing Bariatric Surgery. Gastroenterology 2015 Sep;149(3):623-34.e8. |
| GSE89632 | 20 NAFL patients and 24 controls | Liver | Microarray | GPL14951 | Arendt BM, Comelli EM, Ma DW, Lou W et al. Altered hepatic gene expression in nonalcoholic fatty liver disease is associated with lower hepatic n-3 and n-6 polyunsaturated fatty acids. Hepatology 2015 May;61(5):1565-78. |
| GSE126848 | 15 NAFL patients and 14 controls | Liver | RNA-seq | GPL18573 | Suppli MP, Rigbolt KTG, Veidal SS, Heebøll S et al. Hepatic transcriptome signatures in patients with varying degrees of nonalcoholic fatty liver disease compared with healthy normal-weight individuals. Am J Physiol Gastrointest Liver Physiol 2019 Apr 1;316(4):G462-G472. |
| GSE130970 | 22 NAFL patients and 6 controls | Liver | RNA-seq | GPL16791 | Hoang SA, Oseini A, Feaver RE, Cole BK et al. Gene Expression Predicts Histological Severity and Reveals Distinct Molecular Profiles of Nonalcoholic Fatty Liver Disease. Sci Rep 2019 Aug 29;9(1):12541. |
| GSE135251 | 51 NAFL patients and 10 controls | Liver | RNA-seq | GPL18573 | Govaere O, Cockell S, Tiniakos D, Queen R et al. Transcriptomic profiling across the nonalcoholic fatty liver disease spectrum reveals gene signatures for steatohepatitis and fibrosis. Sci Transl Med 2020 Dec 2;12(572). |
| GSE61260 | 16 older cases and 16 younger cases | Liver | Microarray | GPL11532 | Horvath S, Erhart W, Brosch M, Ammerpohl O et al. Obesity accelerates epigenetic aging of human liver. Proc Natl Acad Sci U S A 2014 Oct 28;111(43):15538-43. |
| GSE107037 | 13 older cases and 13 younger cases | Liver | Microarray | GPL570 | Bacalini MG, Franceschi C, Gentilini D, Ravaioli F et al. Molecular Aging of Human Liver: An Epigenetic/Transcriptomic Signature. J Gerontol A Biol Sci Med Sci 2019 Jan 1;74(1):1-8. |
| GSE133815 | 12 older cases and 11 younger cases | Liver | Microarray | GPL570 | No relevant published study. |
| GSE183915 | 8 older cases and 9 younger cases | Liver | RNA-seq | GPL24676 | Schreiter T, Gieseler RK, Vílchez-Vargas R, Jauregui R et al. Transcriptome-Wide Analysis of Human Liver Reveals Age-Related Differences in the Expression of Select Functional Gene Clusters and Evidence for a PPP1R10-Governed 'Aging Cascade'. Pharmaceutics 2021 Nov 25;13(12). |
| GSE148849 | 6 TGF-β stimulated HSCs and 6 DMOS stimulated HSCs | Liver | RNA-seq | GPL17021 | Bates J, Vijayakumar A, Ghoshal S, Marchand B et al. Acetyl-CoA carboxylase inhibition disrupts metabolic reprogramming during hepatic stellate cell activation. J Hepatol 2020 Oct;73(4):896-905. |
| GSE136103 | 8 CD45^-^ cases and 9 CD45^+^ cases | Liver | scRNA-seq | GPL20301 | Ramachandran P, Dobie R, Wilson-Kanamori JR, Dora EF et al. Resolving the fibrotic niche of human liver cirrhosis at single-cell level. Nature 2019 Nov;575(7783):512-518. |
| GSE174748 | 2 NAFLD patients and 2 healthy controls | Liver | snRNA-seq | GPL20301 | Filliol A, Saito Y, Nair A, Dapito DH et al. Opposing roles of hepatic stellate cell subpopulations in hepatocarcinogenesis. Nature 2022 Oct;610(7931):356-365. |
| GSE140228 | Non-cancer normal liver tissues | Liver | scRNA-seq | GPL20301 | Zhang Q, He Y, Luo N, Patel SJ et al. Landscape and Dynamics of Single Immune Cells in Hepatocellular Carcinoma. Cell 2019 Oct 31;179(4):829-845.e20. |

(NAFL, Non-alcoholic fatty liver; NAFLD, Non-alcoholic fatty liver disease; RNA-seq, RNA sequencing; scRNA-seq, signal-cell RNA sequencing; snRNA-seq, single-nucleus RNA sequencing)

**Supplementary table 3 Gene lists of the transcriptomic signatures developed for monocyte-macrophage subsets in liver**

| **Gene signatures** | **Gene list** |
| --- | --- |
| CD14^+^Monocytes | S100A8, S100A9, S100A12, VCAN, LYZ, S100A6, FCN1, S100A4, CSTA, MNDA |
| CD16^+^Monocytes | CDKN1C, HES4, TCF7L2, CD79B, MTSS1, LILRA1, TPPP3, LILRA5, CX3CR1, SLC24A4, S1PR4, FAM110A, OAS1, CDH23, MYO1G, SLC2A6, ZNF703, IFITM1, GPBAR1, TESC |
| Macrophages C0 | FCN1, SERPINA1, SAT1, APOBEC3A, IFI30, CD52, NEAT1, LGALS2, CSTA, LST1 |
| Macrophages C1 | FCER1A, CD1C, CD1E, FCGR2B, PKIB, NDRG2, PLD4, IL1R2, CACNA2D3, ADAM28, CCND2, ADAM8, GPAT3, FILIP1L, SLC38A1, HLA-DOA, TMEM109, KCNK6, AXL, RALA |
| Macrophages C2 | LPAR6, TREM2, CLEC10A, FCGR2B, LTC4S, HLA-DQA2, RGS1, FCGR2A, HLA-DQB2, F13A1, LAIR1, GSN, DRAM2, GPR183, SLAMF8, HLA-DOA, SGK1, ADAM28, SDS, ABHD12 |
| Profibrotic Macrophages | OTOA, GPNMB, TREM2, PLA2G7, CD9, CYP27A1, MGLL, ACP5, SDS, VAT1, NPL, SLC38A6, SDSL, SPP1, MITF, CPM, AMDHD2, DNASE2, GLMP, FBP1 |
| Kupffer cells | CD5L, SEPP1, SLC40A1, MARCO, APOE, LGMN, VCAM1, SDC3, CXCL12, CETP, FOLR2, LYVE1, LILRB5, FABP3, RND3, SCD, SMPDL3A, TIMD4, SLC7A8, C2 |

(Macrophages C2, PCT diff>20%, PCT2<0.5 and exclude signature genes of Kupffer cells; Macrophages C0 and CD14^+^Monocytes, PCT diff>0 and adjusted p value<0.05, included top 10 genes; Other signatures, PCT diff>25% and PCT2<0.25)
